# Reversing the Irreversible: miRNA-Targeting Mesyl Phosphoramidate Oligonucleotides Restore Sensitivity to Cisplatin and Doxorubicin of KB-8-5 Epidermoid Carcinoma Cells

**DOI:** 10.3390/biomedicines13123118

**Published:** 2025-12-18

**Authors:** Svetlana Miroshnichenko, Rabia Demirel, Arseny Moralev, Olga Almieva, Andrey Markov, Ekaterina Burakova, Dmitry Stetsenko, Mikhail Maslov, Valentin Vlassov, Marina Zenkova

**Affiliations:** 1 Institute of Chemical Biology and Fundamental Medicine SB RAS, Novosibirsk 630090, Russia; sveta-mira@yandex.ru (S.M.); r.demirel@g.nsu.ru (R.D.); arseniimoralev@gmail.com (A.M.); yakovenko0lya@yandex.ru (O.A.); andmrkv@gmail.com (A.M.); vvv@niboch.nsc.ru (V.V.); 2 Department of Physics, Novosibirsk State University, Novosibirsk 630090, Russia; ekaanabur@yandex.ru (E.B.); d.stetsenko@nsu.ru (D.S.); 3 Institute of Cytology and Genetics SB RAS, Novosibirsk 630090, Russia; 4 Lomonosov Institute of Fine Chemical Technologies, MIREA—Russian Technological University, Moscow 119571, Russia; mamaslov@mail.ru

**Keywords:** resistance to chemotherapy, antisense oligonucleotides, MDR, cancer, cisplatin, doxorubicin, ABCB1, microRNA, miR-17, miR-21, mesyl phosphoramidate

## Abstract

**Background:** Chemotherapy remains one of the main approaches for treating malignant tumors, but repeated exposure to cytostatics leads to multidrug resistance (MDR), increasing tumor aggressiveness and reducing therapeutic efficacy. Identifying adjuvant agents that restore tumor sensitivity to drugs while minimizing toxicity is a cornerstone challenge today. This study aimed to investigate the potential of mesyl phosphoramidate antisense oligonucleotides (µ-ASOs) targeting miR-17, miR-21, and miR-155 as agents for enhancing the efficacy of cisplatin (Cis) and doxorubicin (Dox) in MDR-positive human epidermoid carcinoma KB-8-5 cells. **Methods:** Optimal regimens for the simultaneous application of µ-ASOs and Dox or Cis in KB-8-5 cells, including a concentration-dependent analysis and the type of compound interaction in combinations (synergy/additivity/antagonism), were studied using the MTT assay. Antiproliferative effects of the combinations were assessed using the real-time cell monitoring xCELLigence system. The potential molecular mechanism underlying KB-8-5 cell sensitization to cytostatics was investigated using RT-PCR and Western blot hybridization, supported by bioinformatic reconstruction of the gene network. **Results:** The most effective combinations including µ-ASOs targeting miR-21 and miR-17 together with Cis or Dox demonstrated additive to moderately synergistic effects on KB-8-5 cell viability (HSA synergy score = 4.8–8.7). The co-application of µ-ASOs allowed a 5- to 20-fold reduction in the dose of cytostatics, while maintaining a strong antiproliferative effect of 70–95%. Sensitization of KB-8-5 cells to Cis or Dox following µ-ASO treatment was mediated by a 1.5- to 3-fold decrease in the levels of the well-known MDR marker ABCB1 as well as the newly identified MDR-associated targets ZYX, TUBA4A, and SEH1L. **Conclusions:** miRNA-targeted mesyl phosphoramidate oligonucleotides are effective tools for overcoming resistance to the clinically approved chemotherapeutics cisplatin and doxorubicin. The relationship between miR-21, miR-17, and miR-155 and the novel MDR markers such as SEH1L, TUBA4A, and ZYX was revealed, thereby expanding the current understanding of the molecular mechanisms underlying tumor cell resistance to chemotherapy.

## 1. Introduction

According to the World Health Organization, cancer remains one of the leading causes of mortality worldwide, and the number of newly diagnosed cases is expected to increase exponentially in the coming years [1]. This underscores the urgency and relevance of developing novel, effective therapeutic strategies and reinforcing existing treatment regimens. To date, chemotherapy remains the most therapeutically effective approach to cancer treatment [2]. However, repeated courses of cytostatic drugs inevitably lead to a gradual decline in tumor cell sensitivity and the emergence of a multidrug resistance (MDR) [3]. Consequently, higher doses of chemotherapeutic agents are often required, which in turn exacerbates systemic toxicity and adverse effects in patients as well as increases MDR. Therefore, identifying strategies to preserve or restore tumor cell sensitivity to chemotherapy while reducing systemic toxicity represents a major priority in modern molecular oncology.

A growing body of evidence indicates that small non-coding RNAs—microRNAs (miRNAs)—play a pivotal role in carcinogenesis. Under physiological conditions, miRNAs bind to numerous target mRNAs, sterically blocking their translation and suppressing protein synthesis, thereby regulating fundamental cellular processes [4]. During tumor initiation and progression, the expression and activity of miRNAs are frequently dysregulated, leading to large-scale alterations in intra- and intercellular signaling networks, disease progression, and, importantly, the development of MDR [5].

Among the most well-characterized oncogenic miRNAs implicated in MDR across virtually all known cancer types are miR-17, miR-21, and miR-155. These miRNAs contribute to chemotherapy desensitization through a range of distinct molecular mechanisms.

It is well established that during the development and maintenance of the MDR phenotype, one of the key MDR-associated targets of miR-17 is the Akt signaling regulator phosphatase and tensin homolog (PTEN), as demonstrated in resistant colorectal cancer (CRC) cells [6]. In prostate cancer cells, miR-17—acting as part of the miR-17–92 cluster—has also been found to promote cisplatin resistance through the suppression of additional targets associated with the Akt signaling pathway. Specifically, miR-17 downregulates the expression of an intrinsic Akt pathway inhibitor, leading to enhanced Akt and extracellular signal-regulated kinase 1/2 (ERK1/2) signaling activity [7]. In gastric cancer, resistance to chemotherapeutic agents may arise through miR-17-mediated suppression of death effector domain-containing (DEDD) protein expression [8], whereas temozolomide resistance in glioblastoma has been linked to miR-17-induced upregulation of the autophagy-related gene 7 (ATG7) [8]. Similarly, reduced sensitivity to erlotinib in non-small cell lung cancer (NSCLC) has been associated with miR-17-dependent overexpression of enhancer of zeste homolog 1 (EZH1), a member of the EZH family implicated in drug resistance across multiple malignancies [9]. Importantly, among the three oncogenic miRNAs discussed, miR-17 is most frequently reported to mediate chemoresistance via regulatory interactions with long non-coding RNAs (lncRNAs). Such regulatory axes controlling chemosensitivity have been described in both multiple myeloma and colorectal cancer, where the loss of sensitivity to bortezomib and 5-fluorouracil occurs through the lncRNA DUBR–miR-17–TRFC [10] and lncRNA HOTAIR1–miR-17–BTG Anti-Proliferation Factor 3 (BTG3) [11] signaling pathways, respectively.

Studies investigating the role of miR-21 in the development and maintenance of the MDR phenotype have shown that, similar to miR-17, miR-21 primarily regulates tumor cell sensitivity to cytostatic agents through modulation of the Akt signaling pathway, particularly via the expression of the tumor suppressor PTEN. Targeted downregulation of PTEN by miR-21 has been shown to underlie resistance to doxorubicin in hepatocellular carcinoma [12] and triple-negative breast cancer [13], to CHOP therapy in leukemia, and to cisplatin in gastric cancer and neuroblastoma [14]. Another frequently reported miR-21 target in MDR-positive tumor cells is P-glycoprotein, which mediates the efflux of cytotoxic drugs. Sustained miR-21-dependent overexpression of P-glycoprotein, encoded by ABCB1, has been documented in several subtypes of breast cancer resistant to doxorubicin and Adriamycin [13,15]. In addition, upregulation of other members of the efflux transporter family—specifically ABCC3–6, ABCC2–6, and ABCC3/ABCC5—has been identified as a hallmark of miR-21-mediated MDR in renal cell carcinoma cells [6]. miR-21 has also been reported to modulate chemosensitivity through the activation of nuclear factor-κB (NF-κB) signaling in glioblastoma [14]; the suppression of DNA repair proteins, including human DNA MutS homolog 2 (hMSH2), in gastrointestinal cancers [14]; the regulation of Tissue Inhibitor Of Metalloproteinases 3 (TIMP3) expression in hepatocellular carcinoma [16]; and the downregulation of the transcription factors E2F Transcription Factor 1 (E2F1) and Twist in lung adenocarcinoma [17]. Moreover, miR-21 influences the expression of Ras homolog family member B (RhoB), a protein involved in integrin signaling and cell survival in multiple myeloma [9], and modulates the anti-apoptotic protein Bcl-2 in osteosarcoma [7].

In the case of miR-155, the range of MDR-associated targets is remarkably broad and varies across different cancer types. It has been shown that the p53 protein, which exists in a negative feedback loop with this miRNA, contributes to resistance to multiple chemotherapeutic agents in a variety of tumors [18]. In lung adenocarcinoma, resistance to doxorubicin and cisplatin is associated with increased expression of ATP-binding cassette (ABC) transporters—including ABCB1, ABCC1 and ABCG2, encoding p-glycoprotein, MRP1, and BCRP proteins, respectively, ref. [18]—as well as elevated levels of sirtuin 1 (SIRT1) protein [19]. A frequently reported miR-155 target implicated in chemoresistance is the transcription factor forkhead box O3 (FOXO3a), which in turn regulates key pro-apoptotic downstream proteins such as Bim and p27. Such regulatory cascades have been observed in cisplatin-resistant oral squamous carcinoma cells [20] and in chemoresistant breast cancer cells [21]. In addition, miR-155 has been shown to promote breast cancer resistance by inhibiting its direct target gene, tetraspanin-5 (TSPAN5) [22], and by enhancing the activity of several major signaling pathways, including mitogen-activated protein kinase (MAPK), RHOA, and transforming growth factor beta (TGF-β) [21]. However, it is noteworthy that in some cases of breast cancer, miR-155 can act as a tumor suppressor, counteracting paclitaxel resistance through the downregulation of myeloid differentiation primary response gene 88 (MYD88) [23]. Other miR-155 targets associated with chemoresistance include the gemcitabine-metabolizing enzyme deoxycytidine kinase (DCK) in pancreatic cancer [18] and tumor necrosis factor-α-inducible protein 8 (TNFAIP8) in bortezomib-resistant multiple myeloma cells [24].

Given the strong involvement of miRNAs in the development of the MDR phenotype, researchers are actively exploring the potential of antisense oligonucleotides (ASOs) that inhibit oncogenic miRNAs as tools to overcome MDR and resensitize tumor cells to chemotherapeutic agents. Although clinical trials combining cytostatic drugs with miRNA-targeting oligonucleotides have not yet been initiated, a number of preclinical studies have demonstrated the high efficacy of this approach compared with monotherapy [25].

Previously, our research group comprehensively investigated the antitumor potential of antisense oligonucleotides bearing a novel *N*-mesyl (methanesulfonyl) phosphoramidate modification of the internucleosidic phosphate linkage, targeting highly oncogenic miR-17, miR-21, and miR-155 (µ-ASOs) [26,27,28]. These compounds have proven to be highly promising standalone anticancer agents, exhibiting exceptional nuclease stability, RNase H-activating capability, and strong pro-apoptotic, anti-migratory, and anti-proliferative effects. These µ-ASOs demonstrated potent inhibition of tumor growth and metastasis in vivo without any detectable signs of systemic toxicity opposite to widely used phosphorothioate analogs [26,27].

In the present study, we extended this line of research and, for the first time, evaluated the potential of the developed µ-ASOs as agents capable of overcoming the MDR phenotype when used in combination with first- and second-line chemotherapeutic drugs—cisplatin (Cis) and doxorubicin (Dox). Using MDR-positive human epidermoid carcinoma cells (KB-8-5), we assessed the efficacy of combining µ-ASO and chemotherapies and characterized the mode of their interaction: synergistic, antagonistic, or additive.

Another focus of this work was to elucidate the potential mechanism of KB-8-5 cell sensitisation to cytostatic agents induced by µ-ASO treatment. The analysis included assessment of the modulation of *ABCB1* gene expression, a well-established marker of drug resistance encoding P-glycoprotein [29], as well as novel MDR-associated markers recently identified by our group, including the nuclear pore complex (NUP) component *SEH1L* and the cytoskeletal and extracellular matrix components *ZYX* and *TUBA4A*, respectively [30].

## 2. Materials and Methods

### 2.1. Synthesis of Oligonucleotides

Modified oligonucleotides were synthesized on an automated DNA/RNA synthesizer ASM-800 (Biosset, Novosibirsk, Russia) on a scale of 0.2–0.4 μmol using conventionally protected (A^Bz^, C^Bz^, G^iBu^ and T) 5′-dimethoxytrityl (DMTr) 2′-deoxyribonucleotide β-cyanoethyl-*N*,*N*-diisopropyl 3′-phosphoramidites and 1000 Å Controlled Pore Glass (CPG) supports loaded with the respective protected deoxyribonucleosides (Sigma-Aldrich, St. Louis, MO, USA) according to the protocols described earlier [26]. All oligonucleotides used in the study are listed in Table 1.

### 2.2. Cell Lines

Human epidermoid carcinoma KB-3-1 cells were purchased from the Russian Culture Collection (Institute of Cytology of the Russian Academy of Sciences, Saint Petersburg, Russia), and its subtype KB-8-5 cells with an MDR phenotype were generously donated by Prof. M. Gottesman (National Institutes of Health, Bethesda, MD, USA). The KB-3-1 and KB-8-5 cell lines were cultured in Dulbecco’s Modified Eagle’s Medium (DMEM) (Sigma-Aldrich, St. Louis, MO, USA) supplemented with 10% fetal bovine serum (FBS) (Thermo Fisher Scientific, Waltham, MA, USA), 1% antibiotic-antimycotic solution (100 U/mL penicillin, 0.1 μg/mL streptomycin, 0.25 μg/mL amphotericin B) (MP Biomedicals, Seven Hills, Australia). KB-8-5 cells were cultivated in the presence of 300 nM vinblastine, that was eliminated 24 h before seeding of cells. Cells were incubated at 37 °C in a 5% CO_2_.

### 2.3. Transfection of Tumor Cells with ASOs

Transfection of KB-8-5 cells was performed using 2X3-DOPE liposomes [31]. μ-ASOs at a concentration of 10–120 nM and liposomes 2X3-DOPE were mixed at an N/P ratio of 4/1 in the serum-free medium Opti-MEM (Thermo Fisher Scientific, Waltham, MA, USA), at equal volumes (80 µL for a 6-well plate, 12.5 µL for a 96-well plate) pre-incubated for 20 min at room temperature, and added to KB-8-5 cells, followed by incubation at 37 °C in a humidified atmosphere with 5% CO_2_ for 4 h in serum and antibiotic-free DMEM. The medium was then replaced with DMEM containing 10% FBS and 1% antibiotic–antimycotic solution, and the cells were further cultivated for 24–96 h.

### 2.4. Cell Viability Test (MTT)

To evaluate the half maximal inhibitory concentration (IC_50_) for chemotherapeutic agents, KB-8-5 cells were seeded into 96-well plates at a density of 8 × 10^3^ cells per well. The following day, cells were treated with Cisplatin (Cis) at a concentration range of 0.05–15 μM or with Doxorubicin (Dox) at a concentration range of 0.5–30 μM. In case of concurrent treatment with µ-ASOs and cytostatics, cells were seeded into 96-well plates at a density of 6–8 × 10^3^ cells per well. The following day, the cells were transfected with oligonucleotides, as described above. Doxorubicin (0–30 µM) or Cis (0–7.5 µM) was added to the cells 4 h, 24 h or 48 h post-transfection. MTT solution at a concentration of 5 mg/mL (Sigma-Aldrich, USA) was added to each well at a dilution of 1:10 (vol.) 48 h after cytostatic administration, and was incubated with the cells for 3 h at 37 °C and 5% CO_2_. Absorbance was measured using the Multiskan RC reader (LabSystems, Atlanta, GA, USA) at 570 nm, and 620 nm was used as the reference wavelength. The type of μ-ASOs and cytostatic interaction was assessed by the HSA model using SynergyFinder software (https://synergyfinder.fimm.fi/ accessed on 28 July 2025). A synergy score < −10 denotes antagonism; −10–10—additive effect; and >10—synergy.

### 2.5. xCelligence Real-Time Analysis of Cell Proliferation and Viability

Proliferation experiments were performed using an xCelligence real-time cell analysis (RTCA) system (ACEA Biosciences, Santa Clara, CA, USA) in an atmosphere of 5% CO_2_ at 37 °C. KB-8-5 cells were seeded at a density of 5 × 10^3^ cells per well on 16-well E-Plates. The following day, the cells were transfected with oligonucleotides, as described above. Dox (6 µM) or Cis (0.375–0.75 µM) was added to the cells 4 h- and 24 h-post-transfection, respectively. Cell proliferation experiments were run for 120 h, and the cell index was monitored every 30 min for the entire experiment duration.

### 2.6. qPCR

For the analysis of the mRNA level of the MDR-associated genes, KB-8-5 cells were seeded into 6-well plates at a density of 10^6^ cells per well. The following day, cells were transfected with oligonucleotides (total concentration 120 nM) as described above. Total RNA was isolated from KB-8-5 cells 24 h, 48 h and 72 h post-transfection with ASOs and from untreated KB-3-1 cells using RiZol Reagent (DIAM, Moscow, Russia), according to the manufacturer’s protocol. cDNA was synthesized using 5 μg of total cellular RNA in 20 μL reaction mixtures containing Reverse transcription buffer (×1) (Biolabmix, Novosibirsk, Russia) and 100 U of M-MuLV-RH-revertase (Biolabmix, Novosibirsk, Russia). Reverse transcription reaction was conducted at 37 °C for 1 h using hexa primer (NNNNNN, where N represents each random nucleotide (A/G/T/C)). The PCR primers used in the study are listed in Table 1. PCR amplification was carried out using BioMaster HS-qPCR SYBR Blue mix (Biolabmix, Novosibirsk, Russia), as described previously [32]. The obtained qPCR data were analyzed by standard Bio-Rad iQ5 v.2.0 software. The ΔΔCt method was used to determine the relative mRNA levels with *HPRT1* and *GAPDH* mRNA levels serving for normalization.

### 2.7. Western Blot

KB-8-5 cells were seeded in 6-well plates at a density of 2.5 × 10^5^ cells per well in serum-free DMEM and, 24 h later, transfected with µ-ASOs at a final concentration of 120 nM in complex with cationic liposomes 2X3-DOPE as described above. Seventy-two hours post-transfection, the cells were collected and lysed in RIPA buffer at a ratio of 3 × 10^5^ cells per 10 µL of buffer. An aliquot of the obtained lysate (10 µL) was mixed with an equal volume of 2× Laemmli sample buffer (Sigma-Aldrich, USA), heated for 10 min at 95 °C, and subjected to protein electrophoresis. Protein separation was performed using 12.5% SDS–PAGE in a buffer containing 25 mM Tris (pH 8.3), 0.25 M glycine, and 0.1% SDS under an electric field of 10 V/cm for 2 h. Following electrophoresis, proteins were transferred onto an Immobilon-P PVDF membrane (Merck, Rahway, NJ, USA) using wet electrotransfer in a buffer containing 47.9 mM Tris (pH 8.3), 38.6 mM glycine, 0.1% SDS, and 10% ethanol, with a constant current of 400 mA for 1 h using a Criterion blotter system (Bio-Rad, Hercules, CA, USA). To prevent nonspecific antibody binding, the membrane was blocked for 1 h in 5% non-fat dry milk (Fluka, Helsinki, Finland) dissolved in TBST buffer containing 137 mM NaCl, 2.7 mM KCl, 10 mM Na_2_HPO_4_, 1.8 mM KH_2_PO_4_ (pH 7.4), and 0.1% Tween 20. The membranes were then incubated overnight at 4 °C with rabbit monoclonal primary antibodies against TUBA4A (DF14454, Affinity, Beijing, China), ABCB1 (E-AB-81589, Elabscience, Wuhan, China), β-actin (PAB340Hu01, Cloud-Clone, Wuhan, China), SEH1L (RMN89801, AntibodySystem, Paris, France), and ZYX (AF7902, Affinity, China), diluted in 5% non-fat milk in TBST at 1:1000 for TUBA4A, β-actin, SEH1L, and ZYX, and 1:4000 for ABCB1. After incubation, the membrane was washed three times for 10 min each with TBST and then incubated for 1 h with goat anti-rabbit secondary antibodies conjugated with horseradish peroxidase (SAA544Rb19, Cloud-Clone, China) diluted to 1:3000. The membrane was subsequently washed three times for 10 min each with TBST. Chemiluminescent detection was performed using the Excellent Chemiluminescent Substrate (ECL) Detection Kit (Elabscience, China) and visualized with the iBright 1500 Imaging System (Invitrogen, Carlsbad, CA, USA). Band intensity analysis was carried out using Gel Analyzer software v.23.1.1 (Dr. Istvan Lazar Jr. and Dr. Istvan Lazar Sr., UD Faculty of Science and Technology, Institute of Earth Sciences, Hungary). The relative protein level in each sample was calculated as the ratio of the target protein band intensity to that of the reference protein β-actin. The obtained ratios were compared between µ-ASO-treated and control (untreated) KB-8-5 cells.

### 2.8. Rhodamine 123 Accumulation Assay

For the Rhodamine 123 (Rho123) accumulation assay, KB-8-5 and KB-3-1 cells were plated in a 24-well plate at 10^5^ cells per well. The following day KB-8-5 cells were transfected with µ-ASO (total concentration 120 nM) as described above and incubated for 24–72 h. At 24, 48 or 72 h the medium was changed to DMEM containing Rho123 (5.25 μM), followed by incubation of the cells under standard conditions for 30 min. After being washed twice with ice-cold PBS and detached with the TrypLE Express reagent (Gibco, Grand Island, NY, USA), the cells were collected, resuspended in fresh medium, and intracellular Rho123 fluorescence was measured with a NovoCyte Flow Cytometer (ACEA Biosciences, San Diego, CA, USA). Ten thousand events were collected for each sample.

### 2.9. Reconstruction and Analysis of miRNA-Target Networks

Experimentally validated target genes of miRNAs were retrieved from the miRTarBase v. 9.0 database (Human) using the CyTargetLinker v. 4.1.0 plugin and visualized with Cytoscape v. 3.10.4. MDR-associated miRNA targets were selected according to their co-occurrence with the term “chemotherapy” in the MEDLINE database using GenCLiP3 (http://cismu.net/genclip3/analysis.php, accessed on 4 November 2025). Terms were considered related if the number of articles was greater than or equal to five. The interactions between retrieved target genes and markers (*SEH1L*, *TUBA4A*, *ZYX*, *ABCB1*) were reconstructed using the STRING plugin (v2.2.0) with a confidence score > 0.6 and no additional interactions.

### 2.10. Statistics

The data obtained were analyzed using a two-tailed unpaired Student’s *t*-test or one-way ANOVA, followed by Tukey’s test, using STATISTICA v. 10. Statistical significance was considered at *p* < 0.05.

## 3. Results

### 3.1. Optimal Scheme of Concurrent Application of µ-ASO Targeted to miR-17, miR-21, and miR-155 with Cytostatics (Cis/Dox)

At the initial stage of the study, we assessed the concentrations of the chemotherapeutic agents used, as well as established an appropriate regimen for their application with miRNA-targeting µ-ASOs. The effect of cisplatin (Cis) on the viability of KB-8-5 cells exhibiting the MDR phenotype was investigated across a concentration range of 0.05–15 µM, while doxorubicin (Dox) was tested in the range of 0.5–30 µM. The analysis was carried out using the MTT assay, which was performed after 48 h of cell incubation with each cytostatic, as this time point is considered optimal for observing the cytotoxic effects of these compounds. It was found that the half-maximal inhibitory concentrations (IC_50_) for KB-8-5 cells were 7.5 µM for Cis and 30 µM for Dox (Figure S1), which is consistent with previously reported data indicating the higher sensitivity of the KB-8-5 subtypes of KB cells to Cis [33].

Subsequently, the efficacy of different co-administration regimens of µ-ASOs and chemotherapeutic drugs was investigated. It is well established that the combination of miRNA-targeting agents with cytostatics can be implemented using two principal approaches: simultaneous or sequential administration [25]. However, the relative efficiency of these regimens often varies significantly between drugs and tumor models, thus requiring optimization for each specific context.

In the present study, we evaluated the efficacy of combined treatment with µ-ASOs and either Cis or Dox using the following three schemes:

1. Simultaneous treatment, in which chemotherapeutic agents were added immediately after a 4 h cell incubation with µ-ASOs/2X3-DOPE lipoplexes needed for transfection (total incubation time with µ-ASO 48 h) (Scheme A, Figure S2a);
2. Pre-treatment for 24 h, where cells were transfected with the oligonucleotide 24 h prior to cytostatic addition (total incubation time with µ-ASO 72 h) (Scheme B, Figure S2b);
3. Pre-treatment for 48 h, where oligonucleotide transfection preceded drug exposure by 48 h (total incubation time with µ-ASO 96 h) (Scheme C, Figure S2c).

**Figure 1:**
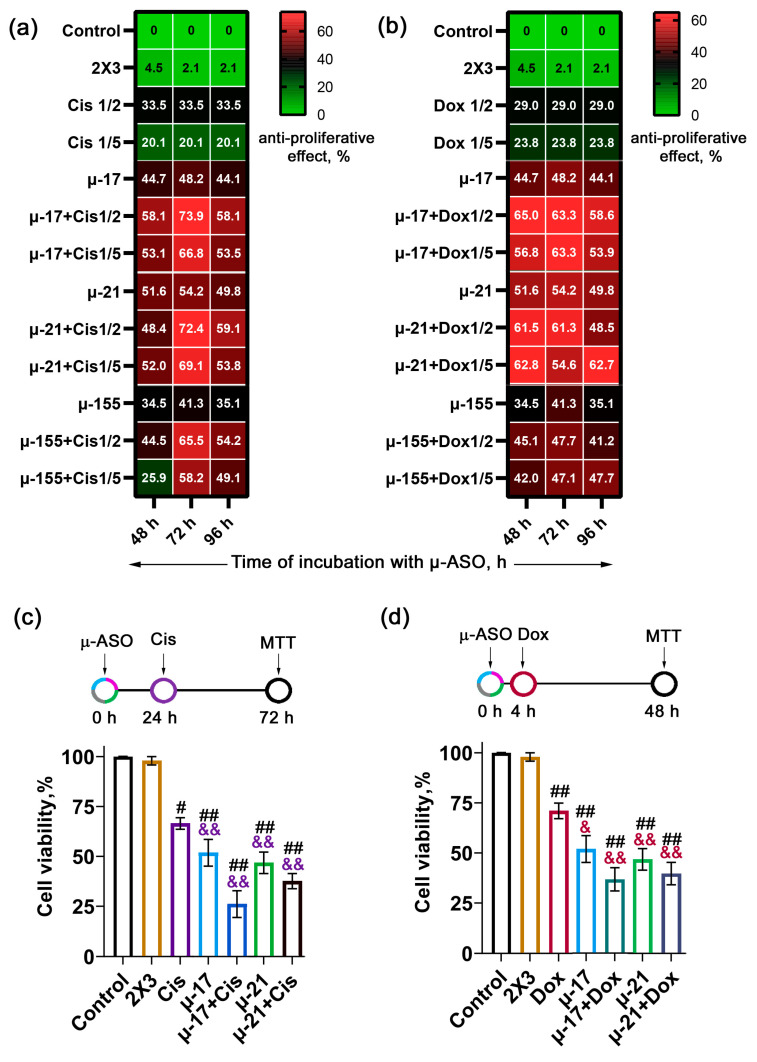
Primary screening of µ-ASOs and cytostatics co-application in KB-8-5 cells. (**a**,**b**) Time-matrix analysis of cell viability inhibition after treatment with µ-ASOs (120 nM) and Cis (**a**) or Dox (**b**) taken at ½ and 1/5 of the IC_50_ dose. Control—intact KB-8-5 cells; 2X3-KB-8-5 cells treated with empty cationic liposomes 2X3-DOPE. Cis or Dox ½ and 1/5-KB-8-5 cells incubated for 48 h with the corresponding cytostatic at ½ and 1/5 IC_50_ doses; µ-ASO + ½Cis/Dox-KB-8-5 cells transfected with µ-ASO (120 nM) followed by 48 h incubation with Cis or Dox at ½ IC_50_ dose; µ-ASO + 1/5 Cis/Dox-KB-8-5 cells transfected with µ-ASO (120 nM) followed by 48 h incubation with Cis or Dox at 1/5 IC_50_. (**c**,**d**) Optimal treatment schedules for (**c**) Cis and (**d**) Dox with graphs, depicting maximal inhibitory effect on KB-8-5 cell viability observed for these schedules (Cis/Dox used at ½ IC_50_ dose). Data show mean viability ± SEM (ANOVA). #, ##—Statistically significant differences from control KB-8-5 cells with *p* ≤ 0.05 and *p* ≤ 0.01, respectively; &, &&—Statistically significant differences from KB-8-5 cells treated with Cis or Dox with *p* ≤ 0.05 and *p* ≤ 0.01, respectively.

Transfection of KB-8-5 cells with µ-ASOs, targeted to miR-17, miR-21 and miR-155 (oligonucleotides µ-17, µ-21 and µ-155, respectively, Table 1) (120 nM concentration in the well) was performed using cationic liposomes 2X3-DOPE at an N:P ratio of 4:1. This concentration of µ-ASO had previously been established as the most effective dose [26]. As a control oligonucleotide, the 21-nt fully µ-modified sequence that has no homology in mammalian genomes-µ-Scramble oligonucleotide (µ-Scr)—was applied (Table 1). Cis and Dox were applied at ½ and 1/5 of the respective IC_50_ values, corresponding to the dosing regimens typically employed in in vivo experiments [34,35,36]. The effect of concurrent treatment was evaluated by the MTT assay after 48 h of cell exposure to the cytostatic agent (Figure 1a,b).

It was found that, under monotherapy conditions, Cis reduced the viability of KB-8-5 cells by 20.1% and 33.5% at concentrations corresponding to 1/5 and ½ of IC_50_, respectively, whereas Dox decreased viability by 23.8% and 29%, respectively (Figure 1a,b, Table S1).

When applied individually, the µ-ASOs demonstrated comparably high efficacy in suppressing KB-8-5 cell survival, with effects of 35–40% for µ-155, 45–48% for µ-17, and 51–54% for µ-21 following 48–96 h of incubation (Figure 1a,b, Table S1).

In turn, the combined treatment of µ-ASOs with Cis or Dox revealed differences in the effectiveness of the combinations depending on the treatment schedule employed.

#### 3.1.1. Regimens of Concurrent Application of μ-ASOs and Cisplatin

The study of the combined application of μ-ASOs with Cis showed that, regardless of the cytostatic concentration (1/5 and ½ of IC_50_), a pronounced peak of tumor cell viability inhibition for all three combination types was observed when Scheme B with 24 h cell pre-incubation with μ-ASOs was applied, while a decrease in efficacy was observed when scheme C was used with pre-incubation time extended to 48 h (Figure 1a, Table S1).

The lowest efficacy was observed for the combination of Cis with μ-155, which suppressed KB-8-5 cell viability by 25.9%, 58.2%, and 49.1% following 48, 72, and 96 h of incubation at 1/5 IC_50_ Cis, and by 44.5%, 65.5%, and 54.2%, respectively, at ½ IC_50_ (Figure 1a, Table S1). In contrast, the combinations with μ-21 and μ-17 showed higher activity when used together with Cis. The application of μ-21 with Cis reduced KB-8-5 cell viability by 52.0%, 69.1%, and 53.8% at 1/5 IC_50_, and by 48.4%, 72.4%, and 59.1% at ½ IC_50_ after 48, 72, and 96 h of incubation, respectively (Figure 1a, Table S1). Similarly, the treatment with μ-17 and Cis resulted in 53.1%, 66.8%, and 53.5% inhibition of cell survival at 1/5 IC_50_ and 58.1%, 73.9%, and 58.1% at ½ IC_50_ after the same time points (Figure 1a, Table S1).

These results indicate that the optimal regimen for the combined application of μ-ASOs and Cis is pre-incubation of cells with the oligonucleotide for 24 h prior to the addition of the cytostatic agent (Scheme B).

Application of Scheme B led to a significant increase in effects of combinations of Cis and μ-ASOs in comparison to monotherapy (Figure 1c). It was shown that concurrent treatment with Cis at ½ IC_50_ and μ-21 or μ-17 resulted in 72.4% and 73.9% inhibition of KB-8-5 cell viability, respectively (Figure 1c). It should be noted that the observed combinative effects were 2.2-fold and 1.5-fold higher than monotherapy with Cis or μ-ASOs alone, respectively (Figure 1c, Table S1).

#### 3.1.2. Regimens of Concurrent Application of μ-ASOs and Doxorubicin

When μ-ASOs were applied with Dox, different patterns of viability suppression were observed. Regardless of Dox concentration (1/5 and ½ of IC_50_), the maximum efficacy of the concurrent treatment was achieved when Scheme A-simultaneous addition of the components was used (Figure S2a, total incubation time with μ-ASO 48 h, Figure 1b), while longer incubation of cells with μ-ASOs (Scheme B and C) did not provide a higher inhibition of cell viability (Figure 1b).

Similarly to Cis, the concurrent application of μ-155 and Dox demonstrated the lowest activity, with a reduction in cell viability to 42–47% (Figure 1b, Table S1). In contrast, the application of μ-17 or μ-21 with Dox showed stronger effects, decreasing KB-8-5 cell viability by 55–62% after 48 h of incubation (Figure 1b, Table S1).

Therefore, it was established that the optimal regimen for co-application of μ-ASO and Dox is Scheme A—simultaneous administration, in which Dox is added immediately after cell transfection with μ-ASOs. Prolonging the incubation time with the oligonucleotide did not further improve the efficacy of the concurrent treatment.

Application of Scheme A allowed a considerable increase in the efficiency of concurrent treatment with Dox and μ-ASOs: the maximal inhibitory effects peaked at 62.8% for μ-21 + Dox (1/5 IC_50_) and 65.0% for μ-17 + Dox (½ IC_50_) (Figure 1d, Table S1). The inhibition of cell viability by these combinations was up to 2.7-fold and up to 1.5-fold more effective than Dox and μ-ASO monotherapy, respectively.

It should be emphasized that, within the present study, the control oligonucleotide µ-Scr, applied either alone or in combination with cytostatics, also exhibited an inhibitory effect on KB-8-5 cell viability, reaching up to 60–67% (Table S1). A similar non-specific effect of µ-Scr monotherapy was previously observed in the KB-8-5 in vivo model, as well [27]. However, it is important to note that µ-Scr has been shown to act through miRNA-independent mechanisms, as evidenced by the absence of changes in the levels of the target miRNAs both in cultured cells and in tumor tissue following µ-Scr administration [27]. Furthermore, our recent data on proteomic profiling of Caco-2 cells after treatment with µ-ASOs used in this study revealed only minimal overlap between the profiles of differentially expressed proteins observed for µ-Scr and each miR-17, miR-21, and miR-155-targeted µ-ASOs, underscoring their distinct mechanisms of action [37]. It is therefore most likely that the observed µ-Scr effects are not directly related to the reversal of cellular MDR phenotype, but rather to an enhanced cytoprotective response of the cells to the administration of modified compounds.

In addition, we observed that in both Cis and Dox combinations, μ-155 exhibited the lowest biological activity. Consequently, our further experiments were focused on evaluating the efficacy of μ-17 and μ-21 in concurrent application with Cis and Dox.

### 3.2. Revealing the Type of Interaction Between µ-ASO and Cytostatics Applied Together on KB-8-5 Cells (Additive/Synergistic Effects)

Further experiments were aimed at analyzing the type of interaction between μ-ASOs and chemotherapeutic agents during co-treatment and at identifying the concentrations of both components that provide the most pronounced synergistic or additive effects on KB-8-5 cell viability.

**Figure 2:**
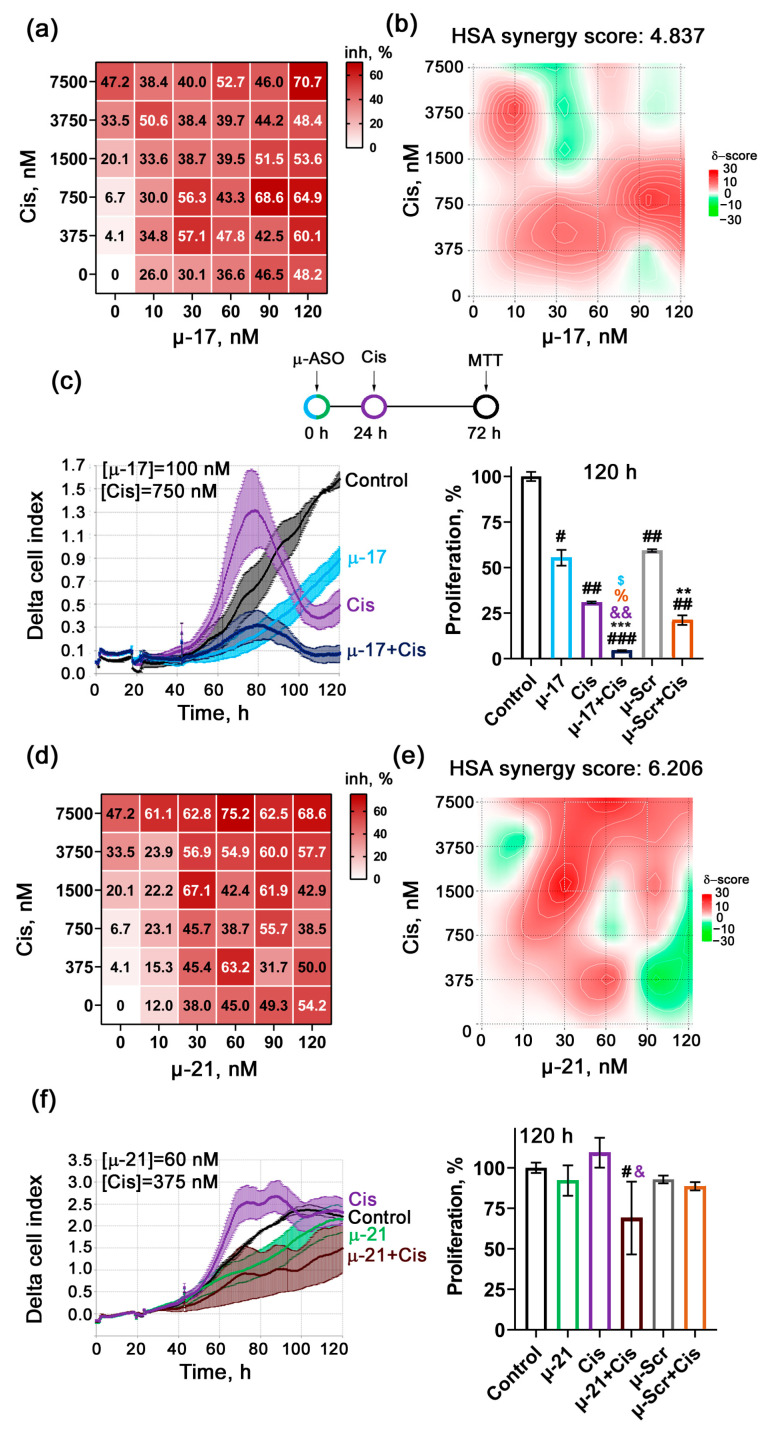
The effect of concurrent application with µ-17 or µ-21 and Cis in KB-8-5 cells. (**a**,**d**) Matrices demonstrating inhibition of KB-8-5 cell growth by different concentrations of Cis and μ-17 (**a**) or μ-21 (**d**) applied alone or in combinations. (**b**,**e**) Heatmaps showing areas with the highest additive-synergetic effects of Cis and μ-17 (**b**) or μ-21 (**e**) on cell growth according to the HSA model. (**c**,**f**) Real-time monitoring of KB-8-5 cell proliferation in the presence of µ-17 (100 nM) + Cis (0.75 µM) (**c**) and µ-21 (60 nM) + Cis (0.375 µM) (**f**) (left panels). Graphs represent proliferation activity of KB-8-5 cells 120 h post-transfection with µ-ASOs (right panels). Control—intact KB-8-5 cells, µ-Scr—KB-8-5 cells transfected with control µ-ASO + 2X3-DOPE liposomes, µ-17 and µ-21– KB-8-5 cells transfected with miR-17- and miR-21-targeting µ-ASOs + 2X3-DOPE, respectively; Cis—KB-8-5 cells incubated with cisplatin; µ-Scr + Cis, µ-17 + Cis and µ-21 + Cis—Kb-8-5 cells incubated with both the respective µ-ASO and Cis. #, ##, ###—statistically significant differences from Control with *p* ˂ 0.05, *p* ˂ 0.01 and *p* ˂ 0.001, respectively; **, ***—statistically significant differences from µ-Scr with *p* ˂ 0.01 and *p* ˂ 0.001, respectively; &, &&—statistically significant differences from Cis with *p* ˂ 0.05 and *p* ˂ 0.01, respectively; %—statistically significant differences from µ-Scr + Cis with *p* ˂ 0.05; $—statistically significant differences from µ-17 with *p* ˂ 0.05.

**Figure 3:**
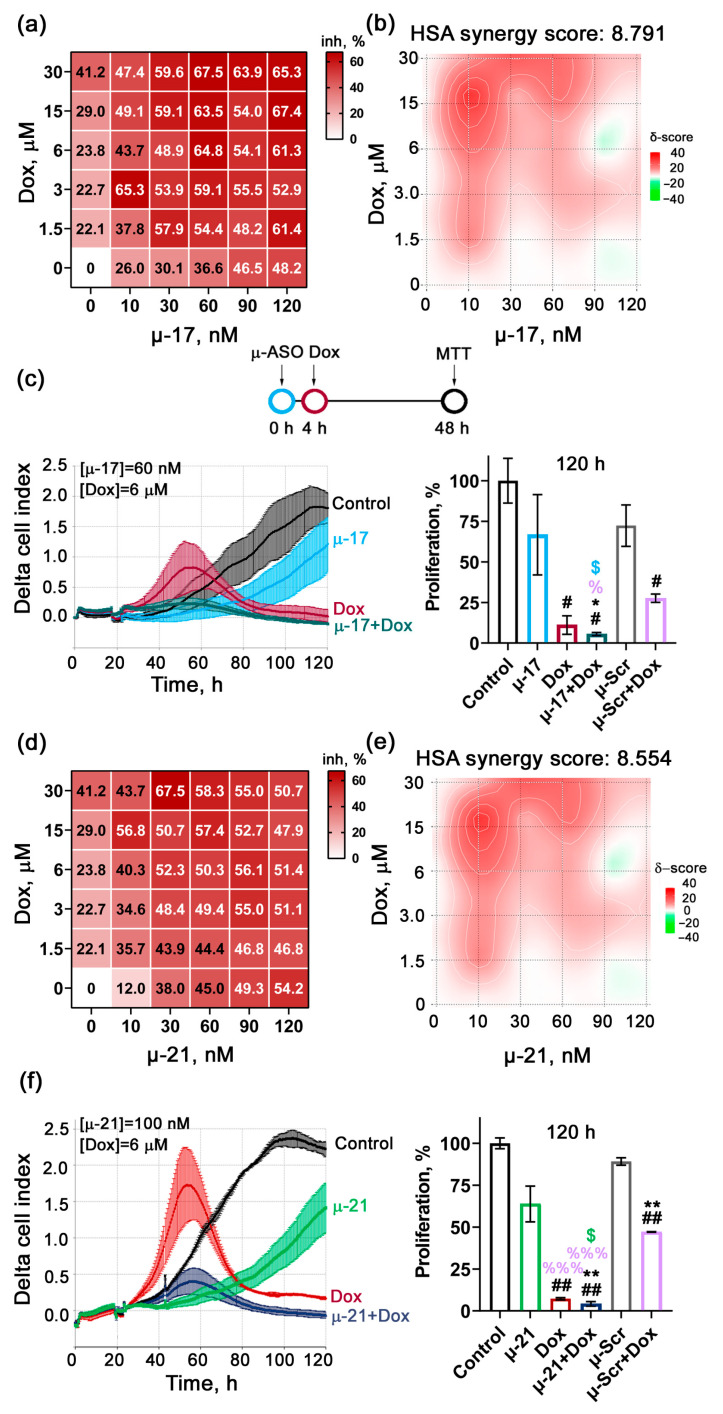
The effect of concurrent application of µ-17 or µ-21 and Dox in KB-8-5 cells. (**a**,**d**) Matrices demonstrating inhibition of KB-8-5 cell growth by different concentrations of Dox and μ-17 (**a**) or μ-21 (**d**) applied alone or in combinations. (**b**,**e**) Heatmaps showing areas with the highest additive-synergetic effects of Dox and μ-17 (**b**) or (**e**) μ-21 on cell growth according to the HSA model. (**c**,**f**) Real-time monitoring of KB-8-5 cell proliferation in the presence of µ-17 (60 nM) + Dox (6 µM) (**c**) and µ-21 (100 nM) + Dox (6 µM) (**f**) (left panels). Graphs represent proliferation activity of KB-8-5 cells 120 h post-transfection with µ-ASOs (right panels). Control—intact KB-8-5 cells, µ-Scr—KB-8-5 cells transfected with control µ-ASO + 2X3-DOPE liposomes, µ-17 and µ-21—KB-8-5 cells transfected with miR-17- and miR-21-targeting µ-ASOs + 2X3-DOPE, respectively; Dox—KB-8-5 cells incubated with doxorubicin; µ-Scr + Dox, µ-17 + Dox and µ-21 + Dox–Kb-8-5 cells incubated with both the respective µ-ASO and Dox. #, ##—statistically significant differences from Control with *p* ˂ 0.05 and *p* ˂ 0.01, respectively; *, **—statistically significant differences from µ-Scr with *p* ˂ 0.05 and *p* ˂ 0.01, respectively; %, %%%—statistically significant differences from µ-Scr + Dox with *p* ˂ 0.05 and *p* ˂ 0.001; $—statistically significant differences from µ-17 or µ-21 with *p* ˂ 0.05.

For this purpose, KB-8-5 cells were transfected with μ-17 or μ-21 at concentrations ranging from 0 to 120 nM pre-complexed with 2X3-DOPE liposomes, followed by treatment with the cytostatic agents: Dox (0–30 μM) added 4 h after transfection, or Cis (0–7.5 μM) added 24 h after transfection. Forty-eight hours after cytostatic treatment, cell viability was assessed using the MTT assay, and concentration matrices were constructed to summarize the effects of mono- and combination treatments (Figure 2a,d and Figure 3a,d). Based on these matrices, heat maps were generated using the SynergyFinder platform to identify the concentrations corresponding to the highest synergistic or additive effects, and synergy scores were calculated according to the Highest Single Agent (HSA) model (Figure 2b,e and Figure 3b,e).

Analysis of the obtained matrices and heat maps revealed that combined treatment with μ-ASOs and cytostatics significantly enhanced antiproliferative efficacy compared to monotherapy. The calculated synergy scores were 4.8 for μ-17 + Cis (Figure 2b), 6.2 for μ-21 + Cis (Figure 2e), 8.8 for μ-17 + Dox (Figure 3b), and 8.6 for μ-21 + Dox (Figure 3e). According to literature data, HSA synergy scores between −10 and +10 indicate additive interactions, while scores in the range of 5–10 may reflect moderate synergism between the agents. Thus, the combined application of studied μ-ASOs and cytostatics demonstrated additive to moderately synergistic suppression of KB-8-5 cell viability.

A more detailed analysis of the matrices and heat maps for all drug combinations showed that several areas of concentration were identified, where additive or moderately synergistic effects on KB-8-5 cell viability were observed (highlighted in dark red on the maps). These areas can be divided into two major types:

1. High μ-ASO concentration (90–120 nM) combined with a 10–20-fold reduced cytostatic concentration compared with monotherapy, while maintaining high overall efficacy (lower right corner of the matrices and heat maps) (Figure 2a,b,d,e and Figure 3a,b,d,e).
2. A two-fold reduction in cytostatic concentration, accompanied by a ten-fold reduction in μ-ASO concentration, which still results in strong antiproliferative effects (upper left region of the matrices and heat maps) (Figure 2a,b,d,e and Figure 3a,b,d,e).

It is noteworthy that, in the context of this study, the most clinically relevant outcome is the ability to significantly reduce cytostatic dosage within the combination regimen. Such an approach could potentially extend the duration of chemotherapy effectiveness before the onset of drug resistance and reduce overall treatment toxicity. In contrast, reducing μ-ASO concentration was not considered essential or critical in this setting, since the applied concentration of 90–120 nM has been repeatedly shown to be highly effective and low-toxic in previous studies [26].

Analysis of the concentration matrix and heat map for the μ-17 + Cis combination revealed that the most pronounced additive effect was achieved at 90–100 nM μ-17 and 0.75 μM Cis, resulting in a 68.6% reduction in KB-8-5 cell viability (Figure 2a,b). Under these conditions, the Cis concentration was reduced tenfold, while the combined treatment produced a 1.5-fold stronger inhibitory effect compared with Cis monotherapy.

Similarly, evaluation of the μ-21 + Cis combination demonstrated that one of the most promising areas of concentration where a moderately synergistic effect was observed is 60 nM μ-21 and 0.375 μM Cis. At these concentrations, cell viability decreased by 63.2%, while the μ-21 and Cis doses were reduced twofold and twentyfold, respectively, compared with corresponding monotherapy (Figure 2d,e).

Assessment of the μ-17 + Dox combination indicated an area of concentration with moderately synergistic interaction at 60 nM μ-17 and 6 μM Dox, resulting in a 64.8% decrease in KB-8-5 cell viability (Figure 3a,b). In this case, μ-17 and Dox concentrations were reduced twofold and fivefold, respectively, relative to their monotherapy concentrations, while the combined treatment achieved a 1.5-fold higher inhibitory effect compared to the maximum Dox dose alone (Figure 3a,b).

Heat map analysis of the μ-21+ Dox combination identified an area of concentration where synergistic effect was observed at 100 nM μ-21 and 6 μM Dox, which resulted in an approximately 50% reduction in KB-8-5 cell viability. Notably, the Dox concentration was reduced fivefold relative to monotherapy, without loss of antiproliferative activity (Figure 3d,e).

Taken together, these findings allowed us to define the optimal concentrations for subsequent experiments investigating the antiproliferative effects of combined μ-ASO and cytostatic treatments on KB-8-5 cells:

μ-17 (100 nM) + Cis (0.75 μM);

μ-21 (60 nM) + Cis (0.375 μM);

μ-17 (60 nM) + Dox (6 μM);

μ-21 (100 nM) + Dox (6 μM).

Under these conditions, the combinations produced the most pronounced additive or moderately synergistic effects on KB-8-5 cell viability.

### 3.3. Uncovering the Biological Effects of Concurrent Application of Cytostatics and μ-ASO in Optimal Concentrations

Particular attention was given to evaluating the antiproliferative effects of drug combinations at concentrations that demonstrated the highest additive or synergistic activity. To this end, KB-8-5 cells were treated with μ-ASOs and cytostatics according to the previously optimized regimens, and cell proliferation was monitored in real time using the xCELLigence detection system for 120 h (Figure 2c,f, Figure 3c,f and Figure S4).

For all four combinations, it was found that low concentrations of cytostatics (0.375 and 0.75 μM Cis, 6 μM Dox) induced a pronounced increase in cell proliferation (a hormetic effect) [38], whereby the cell index doubled compared with untreated KB-8-5 control cells. In contrast, this effect was completely abolished when cytostatics were used in combination with μ-ASOs (Figure 2c,f, Figure 3c,f and Figure S4).

Further analysis of antiproliferative activity revealed that the μ-17 + Cis caused 83% inhibition of cell growth 72 h after transfection, which was 2.1- and 1.7-fold higher in comparison with the control oligonucleotide μ-Scr and μ-Scr + Cis, as well as 2.3- and 1.4-fold higher than monotherapy with Cis or μ-17, respectively (Figure 2c, Figure S3a and Figure S4). The antiproliferative effect of μ-21 + Cis reached 53.8% 72 h post-transfection, which was 1.5-fold higher than that observed for μ-21 monotherapy and 1.3-fold higher than that for μ-Scr + Cis (Figure 2f, Figure S3b and Figure S4). Notably, Cis alone at the tested concentration (0.375 μM) did not inhibit KB-8-5 proliferation but instead stimulated growth of tumor cells (Figure 2f and Figure S3b).

Analysis of Dox-based combinations showed that μ-17 combined with Dox reduced KB-8-5 cell proliferation by 84% at 48 h post-transfection—approximately twofold higher than the effect achieved by Dox alone and μ-Scr + Dox (Figure 3c, Figure S3c and Figure S4). Similarly, the μ-21 + Dox reduced KB-8-5 proliferation by 79.3% at 48 h, corresponding to a 1.5-fold enhancement of therapeutic effectiveness compared with Dox monotherapy and μ-Scr + Dox at the same time point (Figure 3f, Figure S3d and Figure S4).

Importantly, at the final time point (120 h), the combination of μ-17 with Cis achieved 95.6% inhibition of KB-8-5 cell growth compared with the control. In contrast, by this time μ-17 and Cis administered individually suppressed cell growth by 45% and 70%, respectively (Figure 2c, right panel). For all Dox-based combinations (μ-21 + Dox, μ-17 + Dox), complete inhibition of KB-8-5 cell growth was observed (Figure 3c,f). At this time point, the difference from Dox monotherapy was not statistically significant; however, both combinations produced a markedly stronger and statistically significant effect compared with µ-ASO monotherapies, which reduced tumor cell growth by 33–36%.

A unique situation was observed in the case of combined treatment with μ-21 and Cis. At 120 h neither μ-ASO nor the cytostatic alone produced any antiproliferative effect (at 120 h), with Cis even increasing cell growth compared to untreated control (Figure 2f). In turn, μ-21 + Cis exhibited a strong antiproliferative effect leading to a 40% reduction in cell growth relative to control and a 50% reduction relative to Cis alone (Figure 2f).

Taken together, these results clearly demonstrate the significant advantages of combined μ-ASO and cytostatic treatment compared to monotherapy with either compound.

A key finding of the study is the complete suppression of the hormetic effect induced by low cytostatic concentrations when combined with μ-ASOs. Furthermore, the use of μ-ASO–cytostatic combinations reduced the required “working” concentrations of Dox by 10-fold and Cis by 20-fold compared with monotherapy.

### 3.4. Evaluation of Possible Molecular Mechanisms Underlying µ-ASO Action Leading to the Sensitization of KB-8-5 Cells to the Cytostatics

To explore potential mechanisms underlying the sensitization of cells to chemotherapeutic agents, we examined the effects of µ-ASOs targeting miR-17, miR-21, and miR-155 on the mRNA levels of *ABCB1*, *TUBA4A*, *SEH1L*, and *ZYX*, genes previously shown to be associated with the MDR phenotype and upregulated in chemoresistant KB-8-5 cells compared with the parental, chemosensitive KB-3-1 cell line [30]. mRNA levels of these genes were measured at 24, 48, and 72 h post-transfection of KB-8-5 cells with µ-ASOs and compared with their levels in untreated KB-8-5 cells as well as in drug-sensitive KB-3-1 cells.

**Figure 4:**
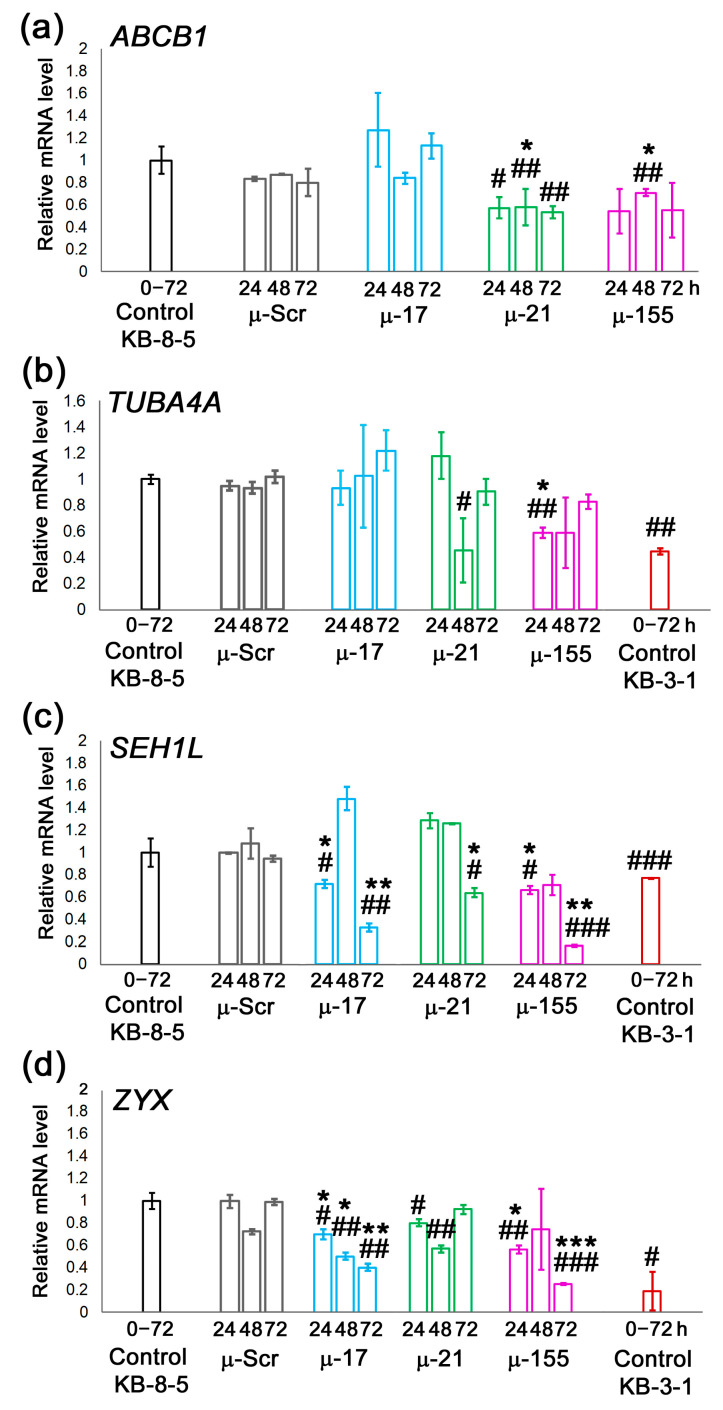
Modulation of MDR-associated gene expression by µ-ASOs targeted to miR-17, miR-21, and miR-155. Relative mRNA expression of (**a**) *ABCB1*, (**b**) *TUBA4A*, (**c**) *SEH1L*, and (**d**) *ZYX* in KB-8-5 cells after treatment with control (µ-Scr) or miRNA-targeted (µ-17, µ-21, µ-155) µ-ASOs pre-complexed with 2X3-DOPE cationic liposomes. Control KB-8-5—intact MDR-positive KB-8-5 cells. Control KB-3-1—parental, drug-sensitive cell line lacking MDR. mRNA levels were quantified by qPCR at 24, 48, and 72 h post-transfection, and normalized to the level of house-keeping genes *HPRT1* and *GAPDH*. *, **, ***—statistically significant differences from µ-Scr with *p* ˂ 0.05, *p* ˂ 0.01, and *p* ˂ 0.001, respectively; #, ##, ###—statistically significant differences from Control KB-8-5 cells with *p* ˂ 0.05, *p* ˂ 0.01, and *p* ˂ 0.001, respectively.

The analysis of *ABCB1* mRNA revealed that µ-17 did not influence its level over the entire incubation period. In contrast, both µ-21 and µ-155 reduced *ABCB1* mRNA levels by 42–46% during the 24–72 h (Figure 4a). Evaluation of *TUBA4A* mRNA showed that µ-17 had no effect on its expression over 24–72 h. µ-21 displayed a distinct pattern, reducing *TUBA4A* mRNA by 55% only at 48 h, whereas levels at 24 and 72 h remained comparable to control cells (Figure 4b). Meanwhile, µ-155 decreased *TUBA4A* mRNA by 41% at 24–48 h with restoration of *TUBA4A* mRNA up to control baseline by 72 h (Figure 4b). Investigation of *SEH1L* mRNA expression showed that µ-21 caused a 36% reduction solely at 72 h (Figure 4c), whereas µ-17 and µ-155 showed a different downregulation profile with reductions averaging ~30% at 24–48 h and reaching 67% and 84%, respectively, at 72 h (Figure 4c). Analysis of *ZYX* mRNA expression revealed distinct dynamics for each µ-ASO (Figure 4d). µ-21 moderately suppressed *ZYX* mRNA by 20% at 24–48 h, with levels returning to baseline at 72 h. µ-17 caused a time-dependent suppression of 30%, 50%, and 60% at 24, 48, and 72 h, respectively. µ-155 produced the most pronounced effect, reducing *ZYX* mRNA by 75% at 72 h (Figure 4d).

Overall, µ-17 had no impact on *ABCB1* and *TUBA4A* but significantly decreased *SEH1L* and *ZYX*, leading to 50–60% reduction in their mRNA levels. µ-21 appears to be capable of enhancing cellular sensitivity to chemotherapeutics by downregulating all examined markers, with the strongest effects observed for *ABCB1* and *TUBA4A* (up to a twofold reduction). µ-155 effectively reduced all examined mRNAs, but its sensitizing effect likely operates primarily through substantial downregulation of *SEH1L* and *ZYX* (up to 75–84%).

It should be noted that treatment with µ-ASOs resulted in highly effective modulation of MDR-associated genes, downregulating their mRNA levels to those observed in parental KB-3-1 cells lacking the MDR phenotype (Figure 4b–d).

To further validate the effects of µ-ASOs on the levels of MDR-associated proteins, we performed Western blot analysis at 72 h post-transfection of KB-8-5 cells with µ-17 and µ-21 (Figure S5). A significant and surprising finding was that µ-17, despite the absence of detectable mRNA level reduction for *ABCB1* and *TUBA4A*, exhibited a pronounced threefold decrease in the levels of these protein markers (Figure S5). Additionally, µ-17 treatment resulted in a twofold reduction in ZYX protein and a threefold reduction in SEH1L, which aligns with the observed decrease in their mRNA levels at the given time point (Figure S5). For µ-21, we found that the effective twofold reduction in the mRNA levels of all markers was generally reflected by a corresponding twofold decrease in their corresponding protein levels, with the exception of ZYX, which showed a 1.5-fold reduction (Figure S5).

**Figure 5:**
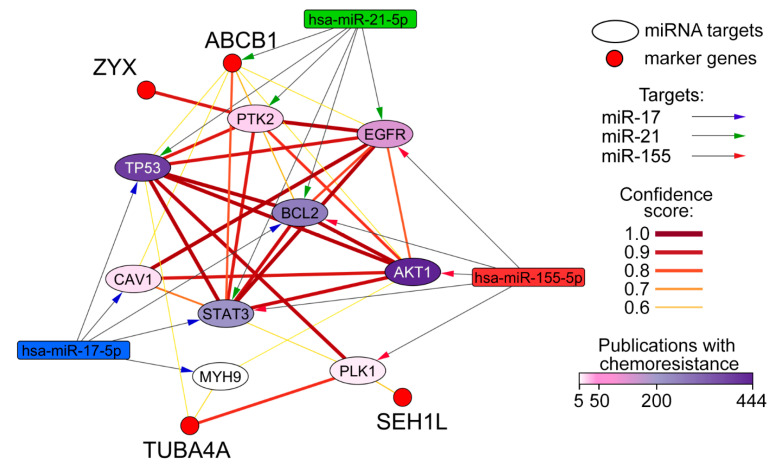
PPI network, demonstrating the potential mechanism of regulation of MDR-associated gene expression by miR-17, miR-21, and miR-155. The PPI network was reconstructed using experimentally validated target genes of miR-17, miR-21, and miR-155 from the miRTarBase v. 9.0 database (Human) associated with the term “chemoresistance” according to the MEDLINE database. The interactions between the retrieved target genes and markers (*SEH1L*, *TUBA4A*, *ZYX*, *ABCB1*) were reconstructed using the STRING plugin (v2.2.0) with a confidence score > 0.6 and no additional interactions. Node details: Red circles represent the MDR-associated markers (*SEH1L*, *TUBA4A*, *ZYX*, *ABCB1*). Oval nodes represent miR-17, miR-21, and miR-155 targets linked to “chemoresistance” term. Node color indicates publication count for each gene, ranging from white (≥5 publications) to dark purple (≤444 publications). Edge (line) details: The color and thickness of edges represent the confidence score of protein–protein interactions, ranging from thin yellow (score 0.6) to thick red (score 1.0). Interaction arrows: Direct regulatory interactions of the miRNAs with their targets are indicated by arrows (dark blue for miR-17, green for miR-21, red for miR-155).

Since the observed effects are predominantly the downregulation of the selected markers, it is likely that they do not represent direct targets of miR-17, miR-21, or miR-155, but instead respond to upstream network rearrangements within the molecular interactomes governed by the corresponding miRNA regulomes. To elucidate this, we reconstructed a fragment of the protein–protein interaction (PPI) network to propose a potential mechanism for the regulation of our markers by the target miRNAs. We curated a pool of previously validated targets of miR-17, miR-21, and miR-155 that are associated with chemoresistance in various cancers and identified the proteins most likely to be connected to our markers of interest (Figure 5).

Our model suggests that the most probable upstream regulator of ZYX is protein tyrosine kinase 2 (PTK2). PTK2 represents a direct target of miR-21 and is linked within one interaction to both miR-17 and miR-155. Specifically, miR-155 may regulate PTK2 via changes in the expression of epidermal growth factor receptor (EGFR), RAC-alpha serine/threonine-protein kinase (AKT1), or signal transducer and activator of transcription 3 (STAT3), while miR-17 could exert its influence through p53 or STAT3, all of which are direct targets of the respective miRNAs (Figure 5).

The proteins TUBA4A and SEH1L showed the smallest number of regulatory proteins in the reconstructed network. TUBA4A expression appears to be modulated by the differential expression of myosin heavy chain 9 (MYH9) (regulated by miR-17), polo-like kinase 1 (PLK1) (miR-155), and p53 (miR-17, miR-21). SEH1L, in turn, is potentially dependent on the expression of PLK1 (miR-155), STAT3 (miR-17, miR-21, and miR-155), and p53 (miR-17, miR-21). It is important to note that TUBA4A and SEH1L may mutually influence each other’s expression, as they are interconnected via a single primary neighbor in the network (Figure 5).

Conversely, the ABCB1 protein emerged as one of the most highly interconnected MDR markers in our network. Although it is a direct target of miR-21, we observed a reduction in its expression at both the mRNA and protein levels. This is most likely attributable to complex feedback loops involving ABCB1 and proteins such as PTK2 (regulated by miR-21), STAT3 (miR-17, miR-21, miR-155), AKT1 (miR-155), and BCL2 (miR-17, miR-21, miR-155), or other regulators not accounted for in this network, which could collectively contribute to its downregulation in the cells (Figure 5).

Thus, we suggest that the potential mechanism underlying the sensitization of KB-8-5 cells to chemotherapeutic agents involves the modulation of both novel targets (TUBA4A, SEH1L, ZYX) and the well-established MDR marker ABCB1. However, one limitation of the study should be noted. Despite observing a significant reduction in the levels of *ABCB1* mRNA and its protein product P-glycoprotein (P-gp)—a key mediator of chemotherapeutic drug efflux—we did not detect a corresponding increase in the intracellular accumulation of rhodamine 123 in KB-8-5 cells after 24–72 h of incubation with µ-ASOs (Figure S6). This apparent discrepancy can likely be attributed to several factors. First, P-gp is a long-living transmembrane protein with a half-life of up to 72 h; therefore, its functionally active membrane-associated form might persist even after a significant reduction in mRNA levels. Second, KB-8-5 cells possess an extremely high level of P-gp—approximately 5- to 10-fold higher than in tumor tissues of patients who have undergone multiple courses of chemotherapy. Consequently, even a threefold depletion of its level may not reach the threshold required for detectable changes in efflux activity. Additionally, other multidrug transporters (e.g., MRP1, BCRP) may partially compensate for the decreased activity of P-gp especially in highly resistant KB-8-5 cells. Taken together, these factors suggest that the functional consequences of P-gp downregulation may lag behind the observed molecular alterations.

## 4. Discussion

In this study, for the first time, we investigated the effects of combined treatment with mesyl-modified antisense oligonucleotides targeted against miR-17, miR-21, and miR-155 (µ-17, µ-21, and µ-155, respectively) together with the cytostatic agents Cis and Dox in human epidermoid carcinoma KB-8-5 cells, which exhibit a pronounced MDR phenotype. In clinical oncology, Cis and Dox are routinely employed as first- and second-line chemotherapeutics for a broad range of malignancies, including oral squamous cell carcinoma and cervical cancer, which, according to various studies, correspond to the phenotype of KB-derived cell lines [39].

### 4.1. Optimization of Combination Regimens

Our results demonstrate that the optimal treatment regimen depends on the specific cytostatic used. In the case of Cis combinations, the most effective schedule involved a 24 h pre-incubation with µ-ASO prior to the addition of the cytostatic (Figure S2b and Figure 1a,c). This effect is most likely attributable to the fact that cisplatin’s cytotoxic action largely depends on the activity of DNA repair systems, which counteract the drug by excising platinum adducts from nucleic acids and preventing cell-cycle arrest [40]. Under the influence of µ-ASO, the levels and activity of repair proteins may decrease; however, due to the inherent delay associated with the RNA interference mechanism, this process likely occurs with a temporal lag. Consequently, the earliest time point at which a pronounced reduction in tumor cell viability is observed upon combined µ-ASO + Cis treatment is approximately 72 h after µ-ASO transfection.

In contrast, the effects of the Dox combinations remained pronounced even when both agents were administered simultaneously (Figure S2a and Figure 1b,c). This may be due to the fact that doxorubicin’s action is less dependent on long-lived DNA repair proteins, and is more closely linked to the expression of short-living pro- and anti-apoptotic proteins, whose levels can rapidly change in response to miRNA-targeted ASOs [41,42]. Furthermore, the marked efficacy of Dox combinations may be explained by the drug’s kinetic profile: it is known that doxorubicin-induced reactive oxygen species (ROS) production peaks within the first 12 h of incubation with tumor cells, whereas prolonged exposure of Dox in cell culture medium accelerates drug degradation [43].

### 4.2. Mechanistic Interplay Between µ-ASO and Cytostatics

The combined strategy employed in this study offers two major advantages: (1) the convergence of multiple molecular targets and pathways via pharmacologically distinct agents, and (2) the potential amplification of effects through overlapping mechanisms of action.

#### 4.2.1. Divergent Effects Exerted by µ-ASOs and Cytostatics

It is known that µ-ASO exert their function through specific hybridization with target miRNAs, followed by RNase H-mediated cleavage of the RNA strand, derepression of a broad set of tumor suppressor proteins, and large-scale remodeling of intracellular signaling cascades [26]. The miRNAs selected for targeting (miR-17, miR-21, miR-155) are strongly implicated in cytostatic resistance. In the MDR context, the most often mentioned validated targets of miR-17, miR-21, and miR-155 are PTEN, PDCD4, p53, RhoB, and ATG7, which at the same time regulate critical cellular processes such as apoptosis, autophagy, and cell cycle progression [6,7,8,13,15]. Inhibition of miRNAs therefore weakens the adaptive potential of cancer cells and increases their susceptibility to cytostatics. In our experiments, sensitization of KB-8-5 cells was associated with µ-ASO-mediated downregulation of both classical MDR markers, such as ABCB1 (encoding P-glycoprotein), and previously unrecognized proteins. Notably, we established for the first time a relationship between miR-17, miR-21, and miR-155 and the proteins ZYX, TUBA4A, and SEH1L, which are not direct miRNA targets but appear to be affected via upstream regulatory cascades. These newly established MDR markers are involved in adhesion (ZYX), cytoskeletal dynamics (TUBA4A), and mitotic spindle assembly as part of the NUP complex (SEH1L) [30].

In contrast to the target-specific action of µ-ASO, cytostatics exert broader, less selective effects. For instance, Cis exhibits cytotoxicity primarily through covalent crosslinking with DNA and sulfur-rich proteins, leading to ROS accumulation, mitochondrial dysfunction, cellular acidosis, apoptosis, and G2 phase arrest [43]. Meanwhile, Dox acts via three main mechanisms: (1) intercalation into DNA and inhibition of transcription, (2) stabilization of topoisomerase II complexes resulting in double-strand breaks and activation of DNA damage response, and (3) generation of free radicals and initiation of oxidative stress [43].

#### 4.2.2. Complementary and Potentiating Interactions Between µ-ASOs and Cytostatic Agents

##### Coordinated Impact on Cell Cycle Regulation and DNA Repair

Despite the significant mechanistic differences between µ-ASO and chemotherapeutics, several mechanisms are likely to underlie the enhanced efficacy observed with µ-ASO/cytostatics co-treatment. For instance, Cis can interact with tubulin, forming crosslinks that disrupt microtubule assembly and induce mitotic arrest [44]. In our study, µ-ASO treatment also led to downregulation of one of the tubulin subform, TUBA4A, suggesting convergent action of both types of compounds on cytoskeletal components.

Furthermore, the potentiation of cisplatin’s effects might be realized through µ-21-mediated downregulation of MutS Homolog 2 (MSH2) protein expression, as previously identified in our proteomic analysis [37]. MSH2 is a core component of the DNA mismatch repair (MMR) system and is critically involved in recognizing DNA-platinum adducts. Consequently, its suppression is likely to impair the MMR system’s efficacy, thereby enhancing the cytotoxic impact of Cis [45].

##### Complementary Modulation of Cellular Metabolism and Translational Acitivity

An additional mechanism that may explain the additive action of µ-ASOs with both cytostatics is their convergent impact on the metabolic state of tumor cells. It is well-established that both Cis and Dox suppress the glycolysis, through direct inhibition of key glycolytic enzymes and by promoting a shift toward oxidative phosphorylation [46]. A similar mechanism of action has been demonstrated for all the miRNA-targeted oligonucleotides applied in this study [37]. Therefore, one potential basis for the additive-to-moderately synergistic effect of our drug combinations is the dual blockade of ATP synthesis, culminating in profound energy deprivation.

Furthermore, it is known that Cis may suppress RNA polymerase II activity through collisions with platinum-DNA adducts [45]. This effect may be enhanced by µ-17, which was recently shown to significantly downregulate RNA polymerase II expression in tumor cells (according to extensive proteomic profiling carried out after µ-ASO treatment of CRC cells) [37]. Such simultaneous effect of µ-ASO and Cis could potentially reinforce protein synthesis inhibition and promote apoptosis.

Finally, the additive influence of Cis and µ-ASOs may be partially attributable to cisplatin’s ability to counteract compensatory adaptation of tumor cells to µ-ASO treatment. We have previously observed that tumor cells respond to µ-ASO administration by upregulating ribosomal biogenesis and translational activity as a survival mechanism [37]. Cis can effectively suppress this response, as it is known to bind directly to ribosomal RNA and ribosomal proteins, thereby imposing a direct blockade on translational machinery [47].

In summary, the observed enhancement in efficacy from combining µ-ASOs with cytostatics likely stems from a coordinated multi-target attack on the cancer cell’s vital systems—including its metabolic, DNA repair, proliferative, and adhesive machinery. This represents a powerful unification of the functional capacities inherent to both therapeutic classes.

### 4.3. Comparison with Previous Studies

It is important to note that the combinations used in this work (miR-17 and miR-21-targeted oligonucleotides + Cis/Dox) have been previously tested on other tumor cell with differently chemically modified oligonucleotide analogs. However, several key advantages of the present study distinguish it from previously published data.

For instance, the majority of earlier reports describe the co-administration of anti-miRs and cytostatic agents to cell lines that do not possess the MDR phenotype, such as mouse colorectal adenocarcinoma CT-26, human breast adenocarcinomas MCF-7 and MDA-MB-231, human cervical cancer HeLa, and human lung adenocarcinoma A549. The use of such models may not fully recapitulate the process of re-sensitizing therapy-resistant cells to chemotherapeutic agents [18,48,49,50]. In contrast, our study utilized the human epidermoid carcinoma KB-8-5 line, which exhibits an extremely high and notoriously challenging-to-overcome MDR phenotype.

Furthermore, a critical methodological advancement of our work is the comprehensive determination of drug interaction types. Unlike any of the previously mentioned studies, we conducted a rigorous analysis involving heat maps and efficacy matrices to precisely characterize the combinatorial effects. This approach provides conclusive evidence of synergism or additivity, moving beyond simplified observation of enhanced cytotoxicity. Our results established the efficacy across 25 different concentration combinations and clearly delineated zones of moderate synergy and additivity for the active compounds.

The application of µ-ASOs as sensitizing agents for Cis and Dox also demonstrates clear advantages in terms of effect magnitude. A comparison of our data with prior results reveals that our mesyl oligonucleotides achieve comparable or superior efficacy in suppressing tumor cell growth. For instance, previously published data demonstrated that a miR-21-targeted oligonucleotide delivered via a DNA nanohydrogel with Dox induced an average 50–60% reduction in cell viability in HeLa cells [50]. A similar effect (56–60% maximal reduction) was reported in oral squamous cell carcinoma CA-27 cells treated with an anti-miR-21 oligonucleotide + Cis, and in MDA-MB-231 breast cancer cells using anti-miR-21 oligonucleotide nanoparticles with Dox [51,52]. A more pronounced effect was achieved using an anti-miR-21 aptamer with Dox in MCF-7 cells, showing ~70% viability suppression up to tenfold more effective than monotherapy [49]. Evaluations of combination therapies have also shown varying degrees of IC_50_ reduction in co-administered drugs. Typically, combined therapy with miR-17-inhibiting constructs + Cis reduces the drug’s IC_50_ by 1.5 to 2.5-fold across various tumor cell types [53], while the use of commercial miR-21 inhibitors (e.g., from Gene Pharma) can reduce the IC_50_ of Dox by up to fourfold [54]. In turn, our work succeeded in achieving a 5- to 20-fold reduction in the required doses of cytostatics without any loss of the antiproliferative effect, which reached 70–95%.

## 5. Conclusions

Taken together, our findings indicate that overcoming MDR requires simultaneous targeting of multiple signaling, repair, and metabolic pathways. The combination of µ-ASO against miR-17, miR-21, and miR-155 with Cis or Dox represents such a multifaceted approach, enabling a substantial drug dosage reduction without compromising efficacy.

Importantly, this study is the first to reveal a link between miR-17, miR-21, and miR-155 and the newly associated MDR-related proteins ZYX, TUBA4A, and SEH1L, expanding the molecular landscape of drug resistance. These results not only strengthen the rationale for using mesyl phosphoramidate antisense oligonucleotides as potent sensitizing agents, but also lay a conceptual foundation for future development of therapeutic regimens to effectively combat highly drug-resistant tumors.

## Figures and Tables

**Table 1:** Oligonucleotides and primers used in the study.

ASO	Sequence 5′-3′
μ-17	C^μ^T^μ^A^μ^C^μ^C^μ^T^μ^G^μ^C^μ^A^μ^C^μ^T^μ^G^μ^T^μ^A^μ^A^μ^G^μ^C^μ^A^μ^C^μ^T^μ^T^μ^T^μ^G
μ-21	T^μ^C^μ^A^μ^A^μ^C^μ^A^μ^T^μ^C^μ^A^μ^G^μ^T^μ^C^μ^T^μ^G^μ^A^μ^T^μ^A^μ^A^μ^G^μ^C^μ^T^μ^A
μ-155	A^μ^A^μ^C^μ^C^μ^C^μ^C^μ^T^μ^A^μ^T^μ^C^μ^A^μ^C^μ^G^μ^A^μ^T^μ^T^μ^A^μ^G^μ^C^μ^A^μ^T^μ^T^μ^A^μ^A
μ-Scr (Scramble control)	C^μ^A^μ^A^μ^G^μ^T^μ^C^μ^T^μ^C^μ^G^μ^T^μ^A^μ^T^μ^G^μ^T^μ^A^μ^G^μ^T^μ^G^μ^G^μ^T^μ^T 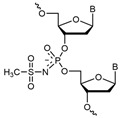 μ-oligonucleotide structure
**PCR Primer**	**Sequence 5**′**-3**′
*ABCB1-F*	AATGGCTACATGAGAGCGGAG
*ABCB1*-R	AATGTTCTGGCTTCCGTTGC
*HPRT*-F	CATCAAAGCACTGAATAGAAAT
*HPRT*-R	TATCTTCCACAATCAAGACATT
*SEH1L*-F	ATAGCGACCAAAGATGTGAG
*SEH1L*-R	CGCCAGACCTGAGAATTATG
*TUBA4A*-F	ATCATTGACCCAGTGCTG
*TUBA4A*-R	CTTGCCATAGTCAACAGAGAG
*ZYX*-F	GCCCTGGACAAGAACTTC
*ZYX*-R	CATCTGCCTCAATCGACAG
*GAPDH*-F	TGCACCACCAAC TGCTTAGC
*GAPDH*-R	GGCATGGACTGTGGTCATGA

^μ^—mesyl phosphoramidate group.

## Data Availability

The original contributions presented in this study are included in the article/Supplementary Materials. Further inquiries can be directed to the corresponding author.

## Supplementary Materials

The following supporting information can be downloaded at: https://www.mdpi.com/article/10.3390/biomedicines13123118/s1, Figure S1: Concentration-dependent inhibition of KB-8-5 cell viability by cisplatin and doxorubicin; Figure S2: Different treatment schemes combining µ-ASO and cytostatics employed in the study; Table S1: The complete dataset showing efficiency of KB-8-5 cell viability inhibition via µ-ASO and cytostatics applied either alone or in combination (%); Figure S3. Real-time monitoring of KB-8-5 cell proliferation after treatment with µ-ASOs and cytostatics (truncated dynamics); Figure S4: Real-time monitoring of KB-8-5 cell proliferation after treatment with µ-ASOs and cytostatics (full dynamics); Figure S5: Relative expression of MDR-associated proteins ABCB1, TUBA4A, SEH1L and ZYX in KB-8-5 cells after treatment with µ-ASO; Figure S6: Investigation of Rhodamine 123 (Rho123) accumulation in KB-8-5 and KB-3-1 cells.

## Abbreviations

The following abbreviations are used in this manuscript:

2X3-DOPE	polycationic lipid 1,26-bis(cholest-5-en-3β-yloxycarbonylamino)-7,11,16,20-tetraazahexacosane tetrahydrochloride 2X3 and helper lipid 1,2-dioleoyl-sn-glycero-3-phosphoethanolamine (DOPE)
ABC	ATP-binding cassette
ABCB1, ABCC, BCRP (ABCG2)	ABC subfamily B member 1, subfamily C members, subfamily G member 2
AKT1	RAC-alpha serine/threonine-protein kinase
anti-miR	Antisense oligonucleotides targeting miR
ASO	Antisense oligonucleotides
ATG7	Autophagy related 7
ATP	Adenosine triphosphate
Bcl-2	BCL2 Apoptosis Regulator
BTG3	BTG Anti-Proliferation Factor 3
cDNA	Complementary deoxyribonucleic acid
CHOP	Cyclophosphamide, hydroxydaunorubicin, oncovin, prednisone
Cis	Cisplatin
CPG	Controlled Pore Glass
CRC	Colorectal cancer
DCK	Deoxycytidine kinase
DEDD	Death effector domain containing
DMEM	Dulbecco’s Modified Eagle’s Medium
DMTr	5′-dimethoxytrityl
DNA	Deoxyribonucleic acid
Dox	Doxorubucun
DUBR	DPPA2 upstream binding RNA
ECL	Excellent Chemiluminescent Substrate
EGFR	Epidermal growth factor receptor
ERK1/2	Extracellular signal-regulated kinase 1/2
EZH1	Enhancer of zeste 1
FBS	Fetal bovine serum
FOXO3	Forkhead box O3
GAPDH	Glyceraldehyde 3-phosphate dehydrogenase
hMSH2	Human DNA MutS homolog 2
HOTAIR1	HOX transcript antisense RNA 1
HPRT1	Hypoxanthine phosphoribosyltransferase 1
HSA	Highest single agent
HS-qPCR	High sensitive quantitative polymerase chain reaction
IC_50_	Half-maximal inhibitory concentration
lncRNA	Long non-coding RNA
mA	Milliampere
MAPK	Mitogen-activated protein kinase
MCF-7	Michigan Cancer Foundation-7
MDA-MB-231	M. D. Anderson Cancer Center Mammary Gland / Breast 231
MDR	Multidrug resistance
miRNA, miR	Micro ribonucleic acid
MMR	Mismatch repair
M-MuLV-RH-revertase	Moloney murine leukemia virus reverse transcriptase with lacked RNase H activity
mRNA	Messenger ribonucleic acid
MRP1	Multidrug-resistance like protein 1
MSH2	MutS Homolog 2
MTT	Thiazolyl blue tetrazolium bromide
MYD88	Myeloid differentiation primary response gene 88
MYH9	Myosin heavy chain 9
NF	Nuclear factor
NSCLC	Non-small cell lung cancer
NUP	Nucleoporins
ON	Oligonucleotide
PAGE	Polyacrylamide gel electrophoresis
PBS	Phosphate-buffered saline
PCR	Polymerase chain reaction
PDCD4	Programmed cell death protein 4
PLK1	Polo like kinase 1
PPI	Protein–protein interaction
PTEN	Phosphatase and tensin homolog
PTK2	Protein tyrosine kinase 2
PVDF	Polyvinylidene fluoride
qPCR	Quantitative polymerase chain reaction
Rho123	Rhodamine 123
RhoA, B	Ras homolog family members A, B
RIPA	Radioimmunoprecipitation assay
RNA	Ribonucleic acid
RNase	Ribonuclease
ROS	Reactive oxygen species
RTCA	Real-time cell analysis
SDS	Sodium dodecyl sulfate
SEH1L	SEH1 like nucleoporin
SIRT1	Sirtuin 1
STAT3	Signal transducer and activator of transcription 3
TBST	TBS and Tween 20 mix
TFRC	Transferrin receptor
TGF-β	Transforming growth factor beta
TIMP3	Tissue inhibitor of metalloproteinase 3
TNFAIP8	Tumor necrosis factor-α-inducible protein 8
TP53	Tumor protein p53
TSPAN5	Tetraspanin-5
TUBA4A	Tubulin alpha-4A
ZYX	Zyxin
ΔΔCt	Delta delta cycle threshold

## References

[1] Siegel R.L., Kratzer T.B., Giaquinto A.N., Sung H., Jemal A. (2025). Cancer Statistics, 2025. CA Cancer J. Clin..

[2] Kaur R., Bhardwaj A., Gupta S. (2023). Cancer Treatment Therapies: Traditional to Modern Approaches to Combat Cancers. Mol. Biol. Rep..

[3] Zhu S., Jin G., He X., Li Y., Xu F., Guo H. (2024). Mechano-Assisted Strategies to Improve Cancer Chemotherapy. Life Sci..

[4] Mohr A.M., Mott J.L. (2015). Overview of MicroRNA Biology. Semin. Liver Dis..

[5] He B., Zhao Z., Cai Q., Zhang Y., Zhang P., Shi S., Xie H., Peng X., Yin W., Tao Y. (2020). MiRNA-Based Biomarkers, Therapies, and Resistance in Cancer. Int. J. Biol. Sci..

[6] Gaudelot K., Gibier J.B., Pottier N., Hémon B., Van Seuningen I., Glowacki F., Leroy X., Cauffiez C., Gnemmi V., Aubert S. (2017). Targeting MiR-21 Decreases Expression of Multi-Drug Resistant Genes and Promotes Chemosensitivity of Renal Carcinoma. Tumor Biol..

[7] Ziyan W., Yang L. (2016). MicroRNA-21 Regulates the Sensitivity to Cisplatin in a Human Osteosarcoma Cell Line. Ir. J. Med. Sci..

[8] Comincini S., Allavena G., Palumbo S., Morini M., Durando F., Angeletti F., Pirtoli L., Miracco C. (2013). MicroRNA-17 Regulates the Expression of ATG7 and Modulates the Autophagy Process, Improving the Sensitivity to Temozolomide and Low-Dose Ionizing Radiation Treatments in Human Glioblastoma Cells. Cancer Biol. Ther..

[9] Abdi J., Jian H., Chang H. (2016). Role of Micro-RNAs in Drug Resistance of Multiple Myeloma. Oncotarget.

[10] Guo T., Zhang F., Wang H., Li H., Xia M., Niu X. (2025). DUBR/MiR-17-3p/TFRC/HO-1 Axis Promotes the Chemosensitivity of Multiple Myeloma. Crit. Rev. Eukaryot. Gene Expr..

[11] Ren T., Hou J., Liu C., Shan F., Xiong X., Qin A., Chen J., Ren W. (2019). The Long Non-Coding RNA HOTAIRM1 Suppresses Cell Progression via Sponging Endogenous MiR-17-5p/ B-Cell Translocation Gene 3 (BTG3) Axis in 5-Fluorouracil Resistant Colorectal Cancer Cells. Biomed. Pharmacother..

[12] Wang W., Huang S., Yuan J., Xu X., Li H., Lv Z., Yu W., Duan S., Hu Y. (2018). Reverse Multidrug Resistance in Human HepG2/ADR by Anti-MiR-21 Combined with Hyperthermia Mediated by Functionalized Gold Nanocages. Mol. Pharm..

[13] Kim E.H., Ryu Y., Choi J., Park D., Lee J.W., Chi S.G., Kim S.H., Yang Y. (2024). Targeting MiR-21 to Overcome P-Glycoprotein Drug Efflux in Doxorubicin-Resistant 4T1 Breast Cancer. Biomater. Res..

[14] Hong L., Han Y., Zhang Y., Zhang H., Zhao Q., Wu K., Fan D. (2013). MicroRNA-21: A Therapeutic Target for Reversing Drug Resistance in Cancer. Expert. Opin. Ther. Targets.

[15] Zhi F., Dong H., Jia X., Guo W., Lu H., Yang Y., Ju H., Zhang X., Hu Y. (2013). Functionalized Graphene Oxide Mediated Adriamycin Delivery and MiR-21 Gene Silencing to Overcome Tumor Multidrug Resistance In Vitro. PLoS ONE.

[16] Yang Q., Wu G. (2022). CircRNA-001241 Mediates Sorafenib Resistance of Hepatocellular Carcinoma Cells by Sponging MiR-21-5p and Regulating TIMP3 Expression. Gastroenterol. Hepatol..

[17] Dong Z., Ren L., Lin L., Li J., Huang Y., Li J. (2015). Effect of MicroRNA-21 on Multidrug Resistance Reversal in A549/DDP Human Lung Cancer Cells. Mol. Med. Rep..

[18] Bayraktar R., Van Roosbroeck K. (2018). MiR-155 in Cancer Drug Resistance and as Target for MiRNA-Based Therapeutics. Cancer Metastasis Rev..

[19] Pang C., Zhang T., Chen Y., Yan B., Chen C., Zhang Z., Wang C. (2023). Andrographis Modulates Cisplatin Resistance in Lung Cancer via MiR-155-5p/SIRT1 Axis. Funct. Integr. Genom..

[20] Sayyed A.A., Gondaliya P., Mali M., Pawar A., Bhat P., Khairnar A., Arya N., Kalia K. (2021). MiR-155 Inhibitor-Laden Exosomes Reverse Resistance to Cisplatin in a 3D Tumor Spheroid and Xenograft Model of Oral Cancer. Mol. Pharm..

[21] Yu D., Lv M., Chen W., Zhong S., Zhang X., Chen L., Ma T., Tang J., Zhao J. (2015). Role of MiR-155 in Drug Resistance of Breast Cancer. Tumour Biol..

[22] Yang L.W., Wu X.J., Liang Y., Ye G.Q., Che Y.C., Wu X.Z., Zhu X.J., Fan H.L., Fan X.P., Xu J.F. (2020). MiR-155 Increases Stemness and Decitabine Resistance in Triple-Negative Breast Cancer Cells by Inhibiting TSPAN5. Mol. Carcinog..

[23] Zhang L., Chen T., Yan L., Xu H., Wang Y., Li Y., Wang H., Chen S., Wang W., Chen C. (2019). MiR-155-3p Acts as a Tumor Suppressor and Reverses Paclitaxel Resistance via Negative Regulation of MYD88 in Human Breast Cancer. Gene.

[24] Rastgoo N., Wu J., Liu A., Pourabdollah M., Atenafu E.G., Reece D., Chen W., Chang H. (2020). Targeting CD47/TNFAIP8 by MiR-155 Overcomes Drug Resistance and Inhibits Tumor Growth through Induction of Phagocytosis and Apoptosis in Multiple Myeloma. Haematologica.

[25] Miroshnichenko S., Patutina O. (2019). Enhanced Inhibition of Tumorigenesis Using Combinations of MiRNA-Targeted Therapeutics. Front. Pharmacol..

[26] Miroshnichenko S.K., Patutina O.A., Burakova E.A., Chelobanov B.P., Fokina A.A., Vlassov V.V., Altman S., Zenkova M.A., Stetsenko D.A. (2019). Mesyl Phosphoramidate Antisense Oligonucleotides as an Alternative to Phosphorothioates with Improved Biochemical and Biological Properties. Proc. Natl. Acad. Sci. USA.

[27] Patutina O., Gaponova S.K., Sen’kova A.V., Savin I.A., Gladkikh D.V., Burakova E.A., Fokina A.A., Maslov M.A., Shmendel’ E.V., Wood M.J.A. (2020). Mesyl Phosphoramidate Backbone Modified Antisense Oligonucleotides Targeting MiR-21 with Enhanced in Vivo Therapeutic Potency. Proc. Natl. Acad. Sci. USA.

[28] Gaponova S., Patutina O., Sen’kova A., Burakova E., Savin I., Markov A., Shmendel E., Maslov M., Stetsenko D., Vlassov V. (2022). Single Shot vs. Cocktail: A Comparison of Mono- and Combinative Application of MiRNA-Targeted Mesyl Oligonucleotides for Efficient Antitumor Therapy. Cancers.

[29] Engle K., Kumar G. (2022). Cancer Multidrug-Resistance Reversal by ABCB1 Inhibition: A Recent Update. Eur. J. Med. Chem..

[30] Moralev A.D., Markov O.V., Zenkova M.A., Markov A.V. (2025). Novel Cross-Cancer Hub Genes in Doxorubicin Resistance Identified by Transcriptional Mapping. Biomedicines.

[31] Kabilova T.O., Shmendel E.V., Gladkikh D.V., Chernolovskaya E.L., Markov O.V., Morozova N.G., Maslov M.A., Zenkova M.A. (2018). Targeted Delivery of Nucleic Acids into Xenograft Tumors Mediated by Novel Folate-Equipped Liposomes. Eur. J. Pharm. Biopharm..

[32] Oshchepkova A., Chernikov I., Miroshnichenko S., Patutina O., Markov O., Savin I., Staroseletz Y., Meschaninova M., Puchkov P., Zhukov S. (2024). Extracellular Vesicle Mimetics as Delivery Vehicles for Oligonucleotide-Based Therapeutics and Plasmid DNA. Front. Bioeng. Biotechnol..

[33] Doherty B., Lawlor D., Gillet J.P., Gottesman M., O’Leary J., Stordal B. Collateral Sensitivity to Cisplatin in KB-8-5-11 Drug-Resistant Cancer Cells. PubMed. https://pubmed.ncbi.nlm.nih.gov/24403508/.

[34] Stojanović M., Lalatović J., Milosavljević A., Savić N., Simms C., Radosavljević B., Ćetković M., Kravić Stevović T., Mrda D., Čolović M.B. (2023). In Vivo Toxicity Evaluation of a Polyoxotungstate Nanocluster as a Promising Contrast Agent for Computed Tomography. Sci. Rep..

[35] Liu J.J., Higgins B., Ju G., Kolinsky K., Luk K.C., Packman K., Pizzolato G., Ren Y., Thakkar K., Tovar C. (2013). Discovery of a Highly Potent, Orally Active Mitosis/Angiogenesis Inhibitor R1530 for the Treatment of Solid Tumors. ACS Med. Chem. Lett..

[36] Kalantari H., Dashtearjandi A.A., Kalantar E. (2009). Genotoxicity Study of Hypiran and Chamomilla Herbal Drugs Determined by in Vivo Supervital Micronucleus Assay with Mouse Peripheral Reticulocytes. Acta Biol. Hung..

[37] Miroshnichenko S., Patutina O., Markov A., Kupryushkin M., Vlassov V., Zenkova M. (2025). Biological performance and molecular mechanisms of mesyl microRNA-targeted oligonucleotides in colorectal cancer cells. Int. J. Mol. Sci..

[38] Furst A. (1987). Hormetic Effects in Pharmacology: Pharmacological Inversions as Prototypes for Hormesis. Health Phys..

[39] Vaughan L., Glänzel W., Korch C., Capes-Davis A. (2017). Widespread Use of Misidentified Cell Line KB (HeLa): Incorrect Attribution and Its Impact Revealed through Mining the Scientific Literature. Cancer Res..

[40] Olaussen K.A., Dunant A., Fouret P., Brambilla E., André F., Haddad V., Taranchon E., Filipits M., Pirker R., Popper H.H. (2006). DNA Repair by ERCC1 in Non-Small-Cell Lung Cancer and Cisplatin-Based Adjuvant Chemotherapy. N. Engl. J. Med..

[41] White S.J., Kasman L.M., Kelly M.M., Lu P., Spruill L., McDermott P.J., Voelkel-Johnson C. (2007). Doxorubicin Generates a Pro-Apoptotic Phenotype by Phosphorylation of EF-2. Free Radic. Biol. Med..

[42] Senichkin V.V., Streletskaia A.Y., Gorbunova A.S., Zhivotovsky B., Kopeina G.S. (2020). Saga of Mcl-1: Regulation from Transcription to Degradation. Cell Death Differ..

[43] Nicoletto R.E., Ofner C.M. (2022). Cytotoxic Mechanisms of Doxorubicin at Clinically Relevant Concentrations in Breast Cancer Cells. Cancer Chemother. Pharmacol..

[44] Makovec T. (2019). Cisplatin and Beyond: Molecular Mechanisms of Action and Drug Resistance Development in Cancer Chemotherapy. Radiol. Oncol..

[45] Fuertes M., Castilla J., Alonso C., Pérez J. (2003). Cisplatin Biochemical Mechanism of Action: From Cytotoxicity to Induction of Cell Death through Interconnections between Apoptotic and Necrotic Pathways. Curr. Med. Chem..

[46] Raudenska M., Balvan J., Fojtu M., Gumulec J., Masarik M. (2019). Unexpected Therapeutic Effects of Cisplatin. Metallomics.

[47] Melnikov S.V., Söll D., Steitz T.A., Polikanov Y.S. (2016). Insights into RNA Binding by the Anticancer Drug Cisplatin from the Crystal Structure of Cisplatin-Modified Ribosome. Nucleic Acids Res..

[48] Gao S., Tian H., Guo Y., Li Y., Guo Z., Zhu X., Chen X. (2015). MiRNA Oligonucleotide and Sponge for MiRNA-21 Inhibition Mediated by PEI-PLL in Breast Cancer Therapy. Acta Biomater..

[49] Khatami F., Matin M.M., Danesh N.M., Bahrami A.R., Abnous K., Taghdisi S.M. (2021). Targeted Delivery System Using Silica Nanoparticles Coated with Chitosan and AS1411 for Combination Therapy of Doxorubicin and AntimiR-21. Carbohydr. Polym..

[50] Lee M., Hwang J., Song Y., Kim S., Park N. (2025). Anti-MiR21-Conjugated DNA Nanohydrogel for Enhanced Cancer Therapy. Biomater. Adv..

[51] Wang W., Songlin P., Sun Y., Zhang B., Jinhui W. (2012). MiR-21 Inhibitor Sensitizes Human OSCC Cells to Cisplatin. Mol. Biol. Rep..

[52] Sun Y., Nie W., Qiu B., Guo X., Zhang J., Wei J. (2021). Inhibition of MicroRNA-17 Enhances Cisplatin-Induced Apoptosis of Human Tongue Squamous Carcinoma Cell. J. Bioenerg. Biomembr..

[53] Wu D.M., Hong X.W., Wang L.L., Cui X.F., Lu J., Chen G.Q., Zheng Y.L. (2018). MicroRNA-17 Inhibition Overcomes Chemoresistance and Suppresses Epithelial-Mesenchymal Transition through a DEDD-Dependent Mechanism in Gastric Cancer. Int. J. Biochem. Cell Biol..

[54] Zhao W., Ning L., Wang L., Ouyang T., Qi L., Yang R., Wu Y. (2021). MiR-21 Inhibition Reverses Doxorubicin-Resistance and Inhibits PC3 Human Prostate Cancer Cells Proliferation. Andrologia.

