# Supplementary material for: Reversing the Irreversible: miRNA-Targeting Mesyl Phosphoramidate Oligonucleotides Restore Sensitivity to Cisplatin and Doxorubicin of KB-8-5 Epidermoid Carcinoma Cells
Source: Biomedicines. 2025 Dec 18;13(12):3118. doi: 10.3390/biomedicines13123118 (PMC12730878; doi:10.3390/biomedicines13123118)
Supplement: Supplementary file 1 [file biomedicines-13-03118-s001.zip › biomedicines-4023296-supplementary.pdf]

Supplementary Materials

Reversing the Irreversible: miRNA-Targeting Mesyl Phosphoramidate Oligonucleotides Restore Sensitivity to Cisplatin and Doxorubicin of KB-8-5 Epidermoid Carcinoma Cells

Svetlana Miroshnichenko [1,†], Rabia Demirel [1,†], Arseny Moralev [1], Olga Almieva [1], Andrey Markov [1], Ekaterina Burakova [2,3], Dmitry Stetsenko [2,3], Mikhail Maslov [4], Valentin Vlassov [1] and Marina Zenkova [1,*]

[1] Institute of Chemical Biology and Fundamental Medicine SB RAS, Novosibirsk 630090, Russia; sveta-mira@yandex.ru (S.M.); r.demirel@g.nsu.ru (R.D.); arseniimoralev@gmail.com (A.M.); yakovenko0lya@yandex.ru (O.A.); andmrkv@gmail.com (A.M.); vvv@niboch.nsc.ru (V.V.)
[2] Department of Physics, Novosibirsk State University, Novosibirsk 630090, Russia; ekaanabur@yandex.ru (E.B.); d.stetsenko@nsu.ru (D.S.)
[3] Institute of Cytology and Genetics SB RAS, Novosibirsk 630090, Russia
[4] Lomonosov Institute of Fine Chemical Technologies, MIREA—Russian Technological University, Moscow 119571, Russia; mamaslov@mail.ru
[*] Correspondence: marzen@1bio.ru or marzen@niboch.nsc.ru; Tel.: +7-383-363-51-60
[†] These authors contributed equally to this work.

Table of consent

Figure S1. Concentration-dependent inhibition of KB-8-5 cell viability by cisplatin and doxorubicin ...…..2

Figure S2. Different treatment schemes combining μ-ASO and cytostatics employed in the study………...2

Table S1. The complete dataset showing efficiency of KB-8-5 cell viability inhibition via μ-ASO and cytostatics applied either alone or in combination (%)……………..……………………………………...3

Figure S3. Real-time monitoring of KB-8-5 cell proliferation after treatment with μ-ASOs and cytostatics (truncated dynamics)…………..……..…………………………………..............................……………...4

Figure S4. Real-time monitoring of KB-8-5 cell proliferation after treatment with μ-ASOs and cytostatics (full dynamics)…………..……..…………………………………..........................……………………..5

Figure S5. Relative expression of MDR-associated proteins ABCB1, TUBA4A, SEH1L and ZYX in KB-8-5 cells after treatment with μ-ASO……..……………………………….............................………………...5

Figure S6. Investigation of Rhodamine 123 (Rho123) accumulation in KB-8-5 and KB-3-1 cells. ……………………...………………….…………………………………..............................…………..………...6

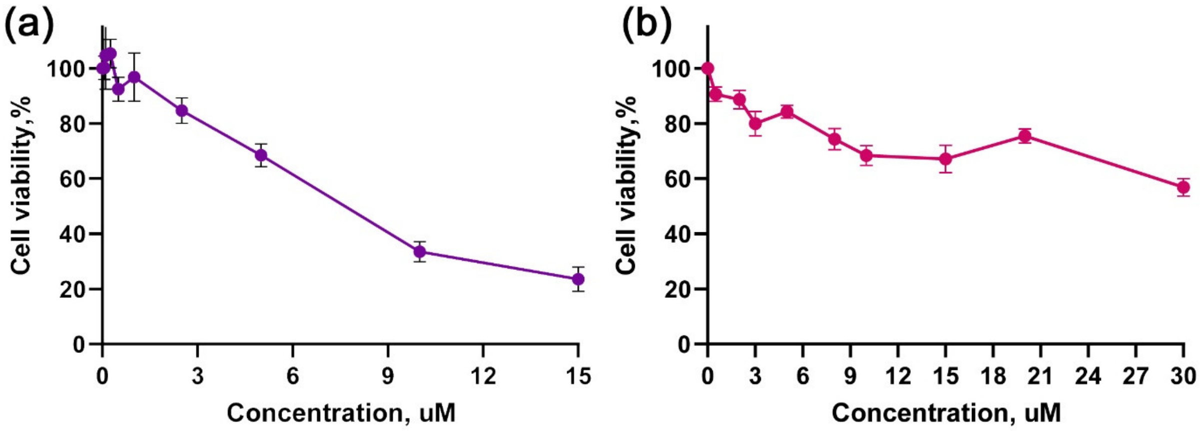

Figure S1. Concentration-dependent inhibition of KB-8-5 cell viability by cisplatin (**a**) and doxorubicin (**b**). KB-8-5 cells were treated with cisplatin (0–7.5 μM) or doxorubicin (0–30 μM) and incubated for 48 h. Cell viability was assessed using the MTT assay. Data are presented as mean ± SEM.

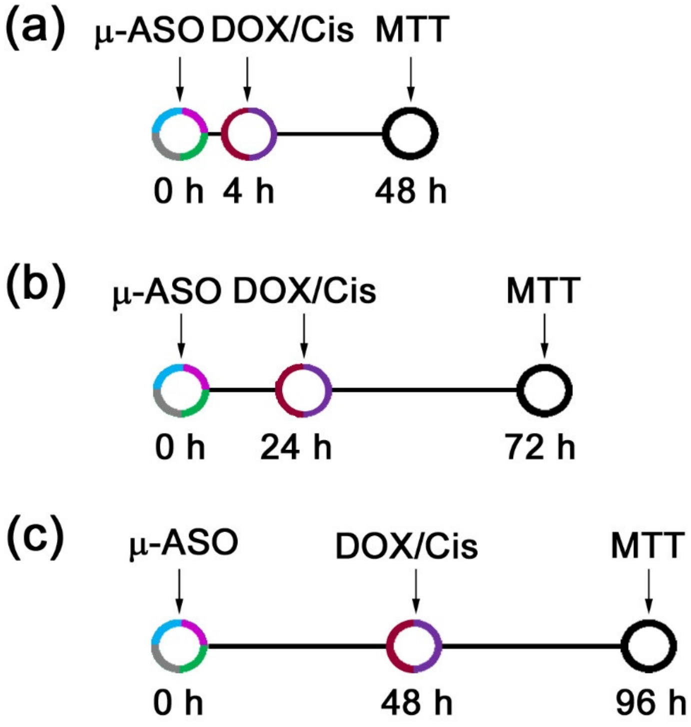

Figure S2. Different treatment schemes combining μ-ASO and cytostatics employed in the study. (**a**) Simultaneous treatment, in which chemotherapeutic agents were added immediately after a 4-hour transfection with the μ-ASOs (total incubation time with μ-ASO: 48 h). (**b**) Concurrent treatment, where cells were transfected with the oligonucleotide 24 h prior to cytostatic addition (total incubation time with μ-ASO: 72 h). (**c**) Concurrent treatment where cells were transfected with the oligonucleotide 48 h prior to cytostatic addition (total incubation time with μ-ASO: 96 h).

Table S1. The complete dataset showing efficiency of KB-8-5 cell viability inhibition via μ-ASO and cytostatics applied either alone or in combination (%).

	Anti-proliferative effect, %		
Compound	Scheme A - Simultaneous treatment (total incubation time with μ-ASO: 48 h)	Scheme B - 24 h pre-incubation with μ-ASO (total incubation time with μ-ASO: 72 h)	Scheme C - 48 h pre-incubation with μ-ASO (total incubation time with μ-ASO: 96 h)
2X3-DOPE	4.5 ± 0.1 %	2.1 ± 2.1 %	2.1 ± 3.0 %
Cis ½ IC50		33.5 ± 2.9 %	
Cis 1/5 IC50		20.1 ± 4.5 %	
Dox ½ IC50		29.0 ± 3.9 %	
Dox 1/5 IC50		23.8 ± 3.7 %	
**μ-Scr**	43.4 ± 5,0 %	41,5 ± 6.2 %	54.9 ± 8.7 %
μ-Scr + Cis ½ IC50	53.3 ± 9.8 %	67.8 ± 2.6 %	61.8 ± 10.0 %
μ-Scr + Cis 1/5 IC50	40.6 ± 6.4 %	63.1 ± 8.5 %	49.6 ± 15.4 %
μ-Scr + Dox ½ IC50	55.7 ± 3.1 %	50.2 ± 5.3 %	55.2 ± 14.8 %
μ-Scr + Dox 1/5 IC50	45.3 ± 6.3 %	53.8 ± 4.2 %	59.6 ± 12.7 %
**μ-17**	44.7 ± 2.4 %	48.2 ± 6.7 %	44.1 ± 16.3 %
μ-17 + Cis ½ IC50	58.1 ± 7.4 %	73.9 ± 6.7 %	58.1 ± 6.1 %
μ-17 + Cis 1/5 IC50	53.1 ± 0.7 %	66.8 ± 3.6 %	53.5 ± 10.1 %
μ-17 + Dox ½ IC50	65.0 ± 0.9 %	63.3 ±5.8 %	58.6 ± 4.0 %
μ-17 + Dox 1/5 IC50	56.8 ± 1.3 %	63.3 ± 3.3 %	53.9 ± 11.8 %
**μ-21**	51.6 ± 3.2 %	54.2 ± 5.4 %	49.8 ± 14.1 %
μ-21+ Cis ½ IC50	48.4 ± 3.5 %	72.4 ± 3.8 %	59.1 ± 10.1 %
μ-21 + Cis 1/5 IC50	52.0 ± 6.7 %	69.1 ± 3.0 %	53.8 ± 8.5 %
μ-21 + Dox ½ IC50	61.5 ± 3.2 %	61.3 ± 5.6 %	48.5 ± 8.6 %
μ-21 + Dox 1/5 IC50	62.8 ± 3.3 %	54.6 ± 5.1 %	62.7 ± 4.2 %
**μ-155**	34.5 ±3.2 %	41.3 ± 7.5 %	35.1 ± 16.4 %
μ-155 + Cis ½ IC50	44.5 ± 6.5 %	65.5 ± 1.1 %	54.2 ± 10.7 %
μ-155 + Cis 1/5 IC50	25.9 ± 7.3 %	58.2 ± 7.6 %	49.1 ± 12.4 %
μ-155 + Dox ½ IC50	45.1 ± 2.3 %	47.7 ± 7.0 %	41.2 ± 4.9 %
μ-155 + Dox 1/5 IC50	42.0 ± 5.8 %	47.1 ± 7.5 %	47.7 ± 12.6 %

Light red color indicates statistically significant differences from corresponding cytostatic, dark red color indicates statistically significant differences from both, corresponding μ-ASO and cytostatic.

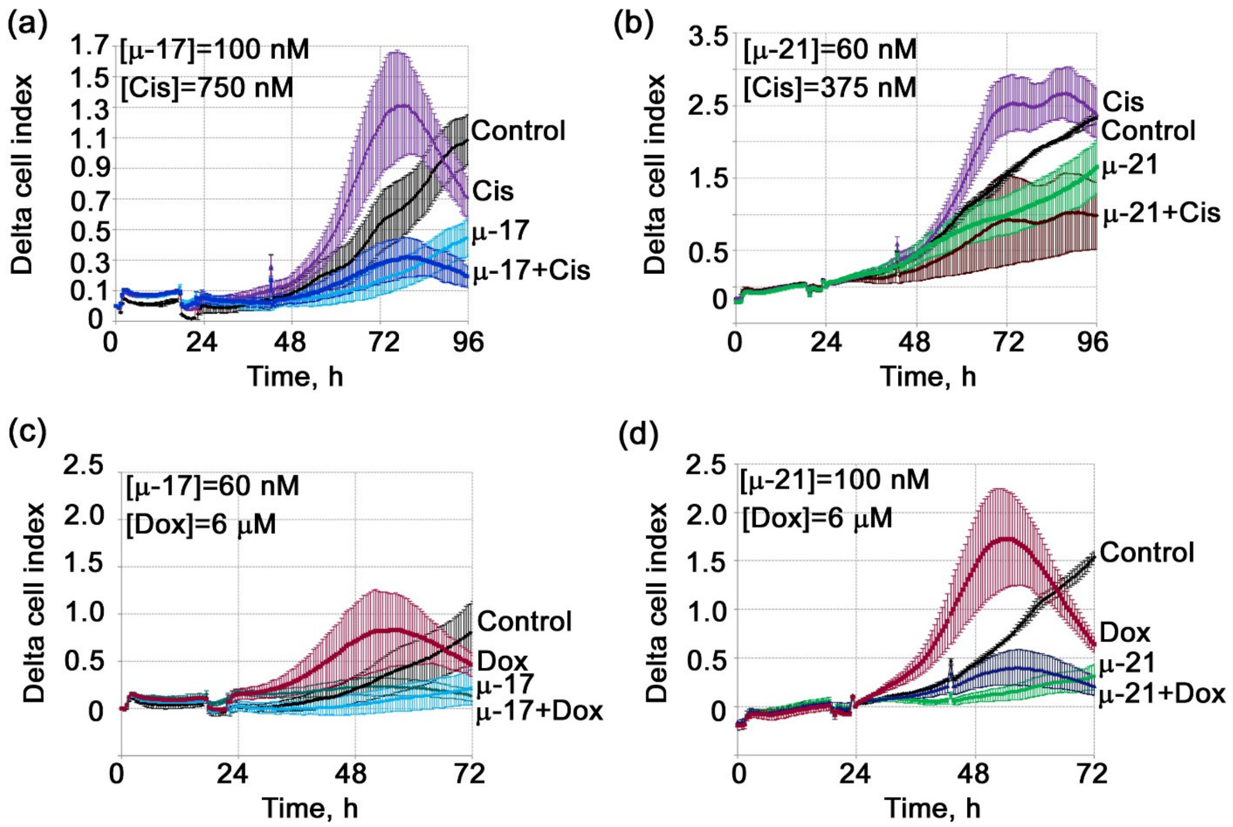

Figure S3. Real-time monitoring of KB-8-5 cell proliferation after treatment with μ-ASOs and cytostatics (truncated dynamics). Real-time proliferation curves of KB-8-5 cells treated with Cis in combination with μ-17 (**a**) or μ-21 (**b**) or with (Dox) in combination with μ-17 (**c**) or μ-21 (**d**) analyzed using the real-time imaging RTCA xCELLigence system. The presented plots correspond to the same experimental settings and treatment conditions as in the main figures (Figure 2 c, f and Figure 3 c, f), but display truncated proliferation dynamics limited 96 h for μ-ASO + Cis combinations (72 h post-transfection with μ-ASO) and to 72 h for μ-ASO + Dox combinations (48 h post-transfection with μ-ASO), reflecting the time points analyzed in the manuscript text. The full 120-h proliferation profiles for the same experiments are provided in the Figure 2 c, f and Figure 3 c, f. Control – intact KB-8-5 cells; μ-21 and μ-17 – cells transfected with miR-21- and miR-17-targeting μ-ASOs, respectively; Dox and Cis – cells treated with respective cytostatics only; μ-21 + drug, and μ-17 + drug – cells treated with both μ-ASO and the corresponding cytostatic.

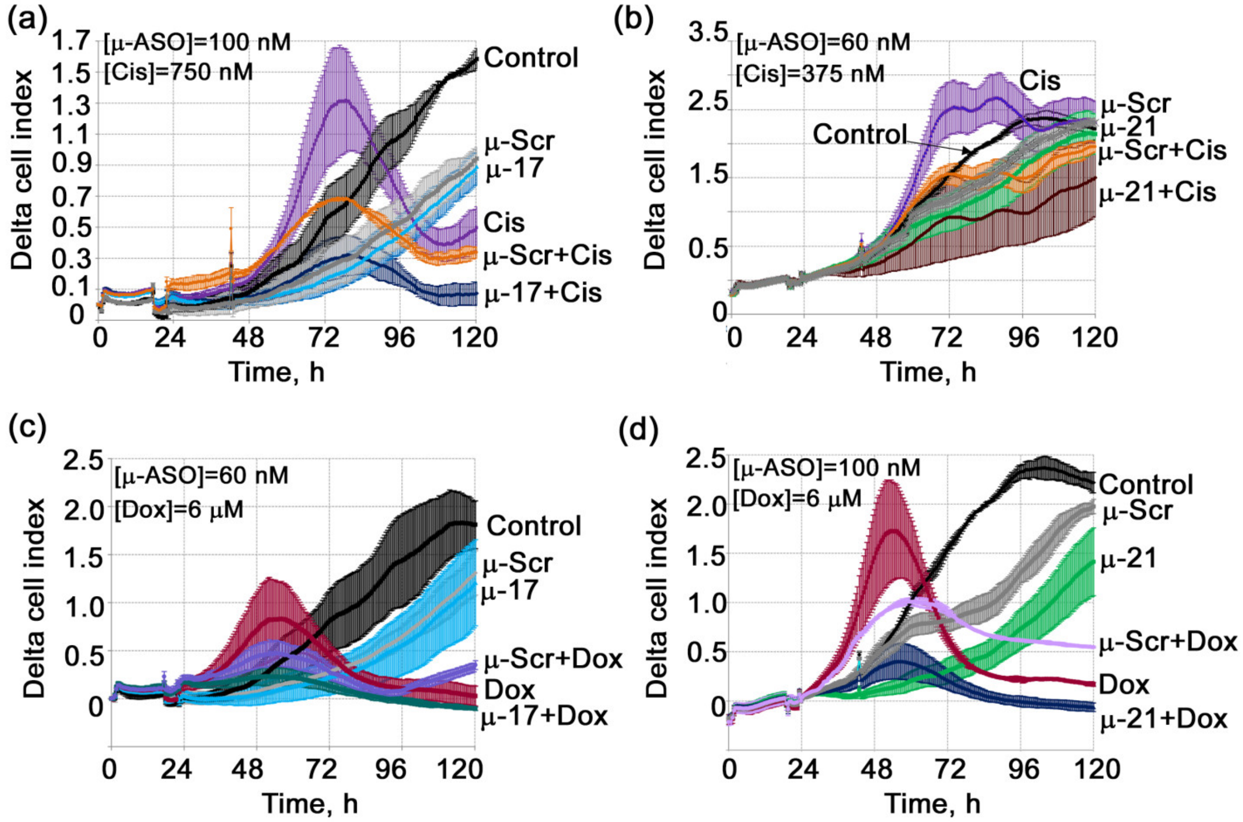

Figure S4. Real-time monitoring of KB-8-5 cell proliferation after treatment with μ-ASOs and cytostatics (full dynamics). Real-time proliferation curves of KB-8-5 cells treated with Cis in combination with μ-17 (**a**) or μ-21 (**b**) or with (Dox) in combination with μ-17 (**c**) or μ-21 (**d**) analyzed using the real-time imaging RTCA xCELLigence system. The presented plots correspond to the same experimental settings and treatment conditions as in the main figures (Figure 2 c, f and Figure 3 c, f), but additionally display cell proliferarion curves for control groups as well, including μ-Scr and μ-Scr + drug. Control – intact KB-8-5 cells; μ-Scr - cells transfected with control scramble μ-ASO; μ-21 and μ-17 – cells transfected with miR-21- and miR-17-targeting μ-ASOs, respectively; Dox and Cis – cells treated with respective cytostatics only; μ-Scr + drug – cells treated with control scramble μ-ASO and the corresponding cytostatic; μ-21 + drug, and μ-17 + drug – cells treated with both μ-ASO and the corresponding cytostatic.

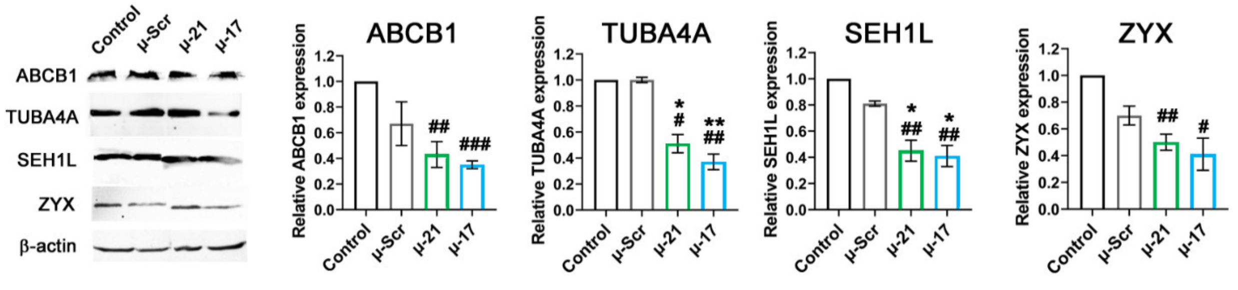

Figure S5. Relative expression of MDR-associated proteins ABCB1, TUBA4A, SEH1L and ZYX in KB-8-5 cells after treatment with μ-ASO. Results of western blot hybridization 72 h post-transfection with μ-ASO. The levels of ABCB1, TUBA4A, SEH1L and ZYX were assessed relative to reference protein β-actin. Control — intact KB-8-5 cells; μ-Scr, μ-21 and μ-17 – KB-8-5 cells treated with control oligonucleotide (μ-Scr) or oligonucleotides targeted to miR-21 and miR-17, respectively, in complex with cationic liposomes

2X3-DOPE in 120 nM concentration. #, ##, ### - statistically significant differences from Control KB-8-5 cells with p-value <0.05, <0.01 and <0.001, respectively; *, ** - statistically significant differences from μ-Scr with p-value <0.05 and <0.01, respectively.

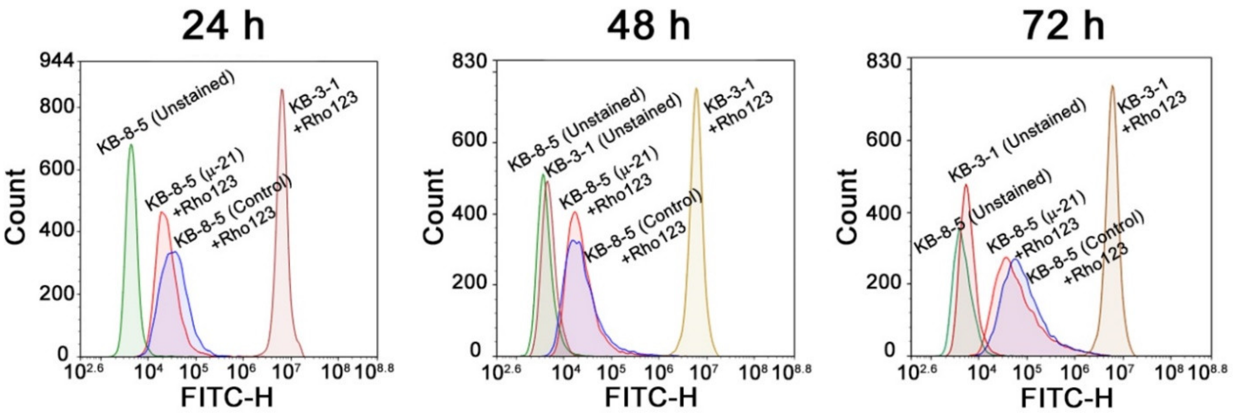

Figure S6. Investigation of Rhodamine 123 (Rho123) accumulation in KB-8-5 and KB-3-1 cells. Rho123 fluorescence intensity in KB-3-1 and KB-8-5 cells after exposure to Rho123 (5.25 μM) for 30 min. The following groups were studied: KB-8-5 and KB-3-1 (unstained) – cells were incubated in the absence of Rho123, KB-8-5 (control) + Rho123 / KB-3-1+Rho123 – control intact cells incubated with Rho123; KB-8-5 (μ-21) + Rho123 – KB-8-5 cells were transfected with μ-21 (120 nM) in complex with 2X3-DOPE and incubated for 24-72 h following by incubation with Rho123.
